# Conserved and particle-specific mitochondrial stress responses drive immunotoxicity of ultrafine particulate matter, black carbon, and polystyrene nanoplastics

**DOI:** 10.3389/fimmu.2026.1993305

**Published:** 2026-10-19

**Authors:** Pradyumna Kumar Mishra, Apoorva Chouksey, Aneha K. Rajan, Ashwani Pathak, Aniket Aglawe, Prakash Punj Dwivedi, Vikas Gurjar, Ravi Prakash Tiwari, Debabrata Dash, Devojit Kumar Sarma, Manoj Kumar, Rajnarayan Tiwari, Rupesh K. Srivastava

**Affiliations:** 1 Division of Environmental Biotechnology, Genetics & Molecular Biology (EBGMB), ICMR National Institute for Research in Environmental Health (NIREH), Bhopal, India; 2 Faculty of Medical Research, Academy of Scientific and Innovative Research (AcSIR), Ghaziabad, India; 3 Division of Environmental Microbiology, Immunology, and Pathology, ICMR National Institute for Research in Environmental Health (NIREH), Bhopal, India; 4 Department of Biotechnology, All India Institute of Medical Sciences (AIIMS), New Delhi, India

**Keywords:** emerging pollutants, environmental toxicants, mitochondrial adaptation, mitochondrial bioenergetics, mitochondrial integrated stress response (mtISR), OMA1-DELE1 signaling, redox signaling

## Abstract

**Introduction:**

Several studies have examined human exposure to ultrafine particulate matter (UFPM), Black carbon (BC), and polystyrene nanoplastics (PS-NPs). However, it remains unclear whether different chemical types of environmental nanoparticles induce a similar mitochondrial stress response or a unique particle-specific response.

**Methods:**

In the present study, we examined the molecular mechanisms underlying nanoparticle-induced mitochondrial stress response and immunotoxicity using human peripheral blood mononuclear cells exposed to UFPM, BC, and PS-NPs under similar experimental conditions. Oxidative stress, mitochondrial adaptation, respiratory chain integrity, mitochondrial integrated stress response, inflammatory signaling, and systems-level interactions between molecules were analyzed through the evaluation of the expression of NRF2, HIF-1α, PGC-1α, TFAM, OMA1, DELE1, mitochondrial ND1, Complex I-V, NF-κB, TNF-α, and NLRP3, and the use of principal component analysis, hierarchical clustering, and correlation networks.

**Results:**

All three nanoparticle exposures elicited oxidative stress and mitochondrial stress-related responses with distinct kinetics and mechanisms. UFPM mostly induced an acute antioxidant response and mitochondrial adaptation; BC led to sustained mitochondrial dysfunction and sustained induction of OMA1 and DELE1 transcripts, consistent with an OMA1-DELE1 associated mitochondrial stress response and inflammation; while PS-NPs induced low but sustained mitochondrial adaptation, along with mitochondrial biogenesis and stress responses. Based on the systems-level analysis, oxidative stress, mitochondrial adaptation, mitochondrial ISR, and inflammation appear to be highly correlated molecular pathways independent of the physicochemical properties of nanoparticles, while the coordinated expression of OMA1 and DELE1 was linked with mitochondrial stress response and inflammation, and the OMA1-DELE1 pathway might be considered as a possible regulatory module for future validation.

**Discussion:**

Overall, we found that activation of the mitochondrial stress response is a common pathway underlying the cytotoxic effects of different chemical nanoparticles. The results of the present study provide new understanding of the molecular pathways responsible for the immunotoxic effects of nanoparticles and identify OMA1, DELE1, NRF2, PGC-1α, TFAM, ND1, and Complex I-V as possible biomarkers.

## Graphical Abstract

**Figure:**
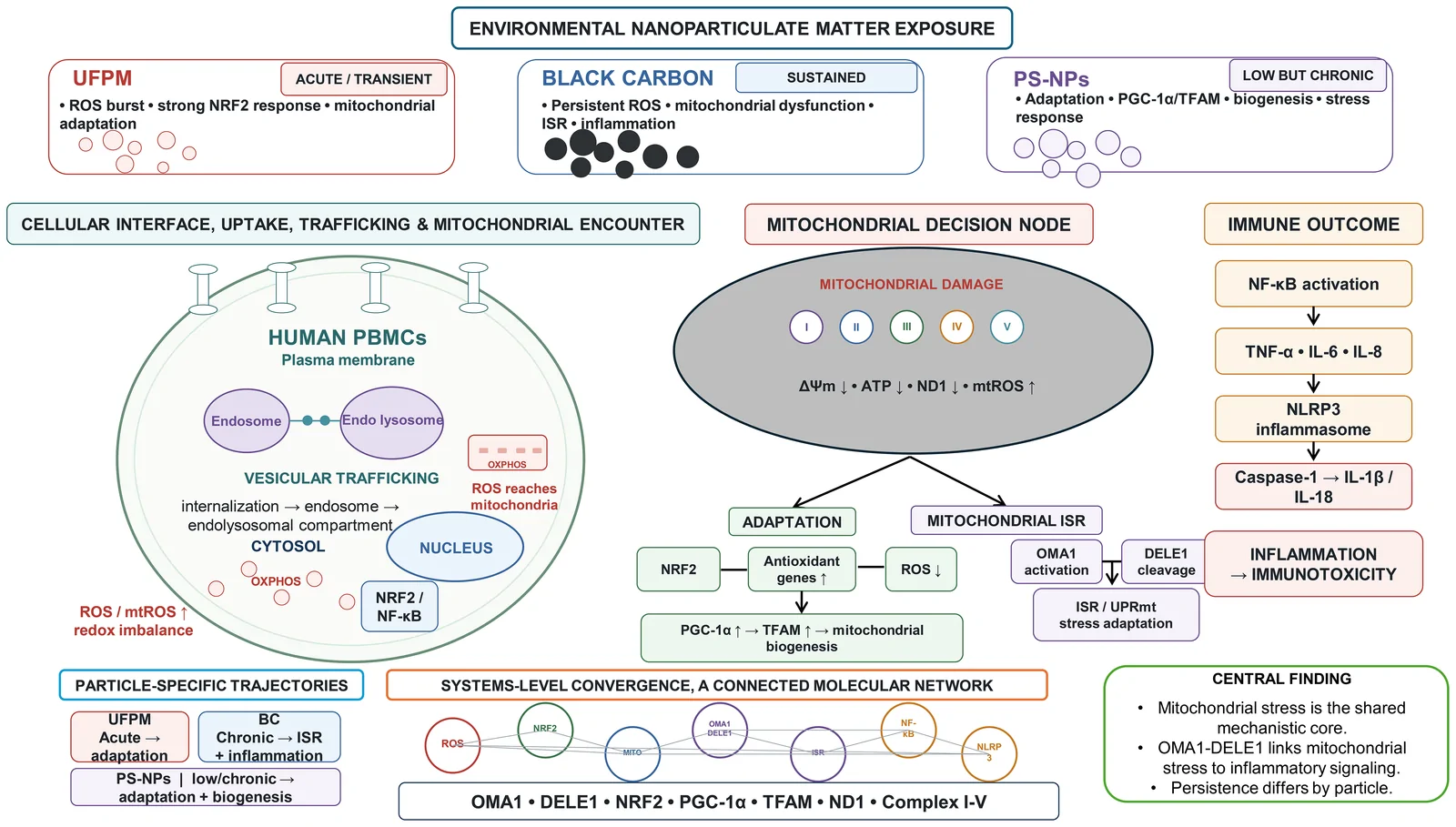

## 1 Introduction

Humans are constantly exposed to nano-sized materials such as ultrafine particulate matter (UFPM), black carbon (BC), and polystyrene nanoplastics (PS-NPs). Nano-sized particles are present in current pollution levels and have been recognized as probable sources of immunotoxicity and inflammation. Even though they exist together in real-world exposure settings of urban air, indoor spaces, and occupational sites, they are chemically unique molecular exposure paradigms that differ substantially in environmental source, chemical composition, surface chemistry, physicochemical properties, cellular uptake and intracellular transport, as well as biological durability (1, 2). As recognition of their toxic effects on human health has increased, most research to date on UFPMs, BC, and PS-NPs has been conducted independently, using varied biological systems and biomarkers. It remains unclear whether chemically different nanoparticles converge on similar mitochondrial stress-response pathways while still showing particle-specific biological responses.

Transition metal ions drive UFPM toxicity, redox cycling, and Fenton reactions, generating oxidative stress, ROS, and mitochondrial damage (3, 4). On the other hand, BC contains PAHs and transition metals that increase its chemical stability; therefore, BC is prone to cellular uptake and cellular actions such as lysosomal dysfunction, metabolism, and inflammation (5, 6). NP-induced toxicity is associated with the rapid formation of a dynamic biomolecular corona (7, 8). Despite the various upstream events that initiate cellular stress induced by the three types of environmental nanoparticles, diverse physicochemical signals converge on mitochondria, the organelle responsible not only for cell bioenergetics but also for integrating metabolic, redox, and inflammatory signaling.

Although awareness of the importance of mitochondrial dysfunction in nanoparticle toxicity is growing, the existing knowledge base has been built on isolated research on individual nanoparticles. Researchers have investigated oxidative stress, mitochondrial DNA (mtDNA) damage, and mitochondrial epigenetics in UFPMs (4, 9), while mitochondrial dynamics, mitophagy, and mitochondrial quality control have been explored in association with BCs (10). In contrast, studies into the molecular mechanisms of NP-induced toxicity have mainly concentrated on redox signaling, the mitochondrial unfolded protein response, and mitochondrial remodeling (8, 11).

There have been several new contributions to the literature on PS-NPs-induced pulmonary toxicity and oxidative stress. Some research indicated that PS-NPs can promote pulmonary fibroblast proliferation and activation, resulting in pulmonary fibrosis due to iron imbalance and cell-cell interaction (12). Nonetheless, current *in vitro* and *in vivo* investigations indicate that PS-NPs exposure leads to oxidative stress, activation of the NRF2/HO-1 signaling pathway, inflammation, mitochondrial dysfunction, and pulmonary toxicity (13). These results also confirm the importance of oxidative and mitochondrial stress in the cellular response to PS-NPs exposure and the need to clarify whether similar stress responses are involved across different chemicals. While these studies have contributed immensely to our understanding of individual nanomaterials, they are limited to certain aspects of mitochondria. These results highlight a key research gap: whether chemically distinct nanoparticulate environmental contaminants use the same mitochondrial pathway or follow distinct stress-induced pathways that result in bioenergetic dysfunction and immunotoxicity. Answering such questions requires a system-wide investigation that can concurrently explore oxidative stress, mitochondrial adaptations, mitochondrial genomic integrity, mitochondrial respiration, and inflammatory signaling. To our knowledge, few studies have directly compared these three exposure types under similar experimental conditions using a common panel of mitochondrial and inflammatory biomarkers.

The mitochondria’s adaptive response to environmental nanoparticles spans a spectrum from adaptation to bioenergetic dysfunction to inflammation, depending on the level of damage sustained. Mitochondrial stress may include ROS production, redox imbalance, calcium overload, Δψm dissipation, and oxidative phosphorylation dysfunction. This collection of processes indicates acute mitochondrial stress, which triggers rather than directly causes damage (4, 14, 15). The first step in acute mitochondrial injury is initiating processes that support mitochondrial repair. Modulation of the HIF-1α-PGC-1α-TFAM pathway is an important part of this process.

Impairment of these regulatory mechanisms may drive irreversible mitochondrial dysfunction from reversible mitochondrial stress. This impairment results in Δψm disturbance, failure of oxidative phosphorylation, and oxidative stress of mtDNA. Under these conditions, retrograde signaling and the OMA1-DELE1-ISR pathway occur, allowing cells to adapt to mitochondrial stress (16–18). Nevertheless, the OMA1-DELE1-ISR pathway has recently been identified as a key orchestrator of mitochondrial stress adaptation; however, it remains unclear whether different nanoparticles use these pathways in distinct ways. The stress response of mitochondria is also a continuum from the adaptive response of mitochondria to dysfunctional behavior and inflammation, which can possibly be mediated through pathways related to the activation of NF-κB and NLRP3 inflammasomes (19–23). UFPMs, BC, and PS-NPs can activate mitochondrial stress through different upstream mechanisms that converge on the mitochondria, where common stress-response pathways are activated with NP-specific kinetics.

However, if oxidative stress surpasses the mitochondria’s adaptive responses, it can damage many parts of the electron transport chain, impairing oxidative phosphorylation and energy production. This is because these complexes work in coordination to produce energy and electrons (24, 25). Due to the presence of the genes responsible for producing respiratory chain proteins and complexes, especially Complex I in the mitochondrial genome, oxidative damage causes mitochondrial genotoxicity as well as respiratory dysfunction (26, 27). Peripheral blood mononuclear cells (PBMCs) serve as a biologically relevant *ex vivo* system for understanding the mechanisms by virtue of the fact that they represent the initial circulating immune cells that come into contact with translocated environmental nanoparticles and depend greatly on mitochondria-mediated metabolism for activation, cytokine production, and inflammatory response (28, 29). Thus, we established an exposure comparison model in which PBMCs were subjected to different environmentally relevant nanoparticles with unique chemical compositions under similar experimental conditions to study whether common or particle-specific mitochondrial stress adaptation pathways exist. The following signaling pathways were examined in detail: mitochondrial adaptation pathway signaling (HIF-1α-PGC-1α-TFAM), mitochondrial stress signaling (OMA1-DELE1-ISR), integrity of the respiratory chain (Complex I-V and mtDNA), inflammatory signaling (NF-κB/NLRP3), and integrative molecular and functional analysis of PBMC responses exposed to environmentally distinct nanoparticles. We hypothesized that although UFPMs, BC, and PS-NPs influence mitochondrial homeostasis, all three use a common mitochondrial stress response system, with particle-specific adaptations that lead to distinct molecular signatures and inflammation/immunotoxic effects.

## 2 Materials and methods

PBMCs were obtained from 30 healthy adult donors and exposed to UFPM, BC, and PS-NPs at a common mass concentration of 1 µg/mL for 6, 12, and 24 h (4, 8, 20, 30, 31) following the norms of the Institutional Ethics Committee [INTR-IM-2024-00063 (PKM)]. For molecular, biochemical, mitochondrial, and inflammatory endpoint analyses, cells were assessed after 6, 12, and 24 h of exposure, with an additional 48 h time point for flow-cytometric assessment of cellular oxidative stress and mitochondrial-associated fluorescence. Donor-derived samples were the independent biological replicates and statistical units. We performed qPCR and ELISA in technical triplicates. We first averaged the technical replicates and then analyzed them statistically without treating them as separate biological samples. For comparisons across particle types, we used a common mass-based exposure as the standardization basis. Because the particles had different distributions of size, aggregation status, and surface area, mass concentration does not equal number concentration or surface area concentration. We characterized particle physicochemical properties, including hydrodynamic particle size and zeta potential, by dynamic light scattering (DLS) and zeta-potential analysis, respectively. However, these parameters reflect the particle suspensions used for exposure but may not fully represent their physicochemical properties under cell culture conditions. We measured oxidative stress and mitochondrial Δψm by flow cytometry, and oxidative DNA damage by the Fpg assay (29). We analyzed mRNA levels of oxidative stress-responsive genes and inflammatory pathways, including NRF2, SOD2, HIF-1α, PGC-1α, TFAM, NF-kB p65, NF-kB, and NLRP3, by one-step quantitative real-time PCR. We assessed mitochondrial DNA integrity and electron transport chain (ETC) complex activity using quantitative real-time PCR and activity measurements, respectively (20, 32). We assessed inflammatory cytokine responses relevant to immunotoxicity by measuring IL-1β, IL-6, IL-8, and TNF-α levels by ELISA (4). We presented gene expression results as mean fold-change (log2 ± SEM) based on biological replicates. For datasets considering nanoparticle type and exposure duration, we used two-way ANOVA to assess the effects of nanoparticle exposure, exposure time, and their interaction. If results were statistically significant (p < 0.05), we performed *post-hoc* comparisons between pairs using an appropriate adjustment for multiple testing. We calculated the Pearson correlation coefficient (r) and R-squared, along with their p-values, for biological replicates across particle types and exposure durations. We interpreted these correlation and shared-variance analyses as measures of statistical association and did not use them to infer temporal, mechanistic, or causal relationships between variables. We conducted Principal Component Analysis (PCA), Hierarchical Clustering, and K-Means Clustering using the biological replicate data matrix, and we correlated gene expression with levels and cytokine levels measured across various exposure conditions and time points. We performed statistical analyses in Python (33–35). The complete experimental protocol is given in the **Supplementary Methods**.

## 3 Results

### 3.1 DLS and zeta-potential characterization reveal distinct physicochemical profiles of UFPM, BC, and PS-NPs

We characterized the physicochemical properties of the three environmental nanoparticulate materials in the comparative exposure model using hydrodynamic particle size and zeta potential analysis by DLS. The three particulate preparations showed distinct size-distribution patterns, suggesting different hydrodynamic properties in suspension (**Figures 1A–C**). BC showed a predominant hydrodynamic particle size of 220.2 nm, whereas PS-NPs showed 566.2 nm. Conversely, UFPM showed a heterogeneous particle distribution with centers at approximately 34.1, 566.1, and 903.0 nm. The heterogeneous distribution of UFPM could indicate multiple particle types and/or aggregated UFPMs in the suspension. Zeta-potential analysis showed that the three particulate preparations differ in surface charge properties (**Figures 1D–F**). BC showed a zeta potential of -19.9 mV, while PS-NPs had the least negative zeta potential (-16.3 mV). UFPM exhibited the most negative zeta potential (-26.1 mV). Thus, the three particulate preparations differed in both hydrodynamic size and surface charge.

**Figure 1:**
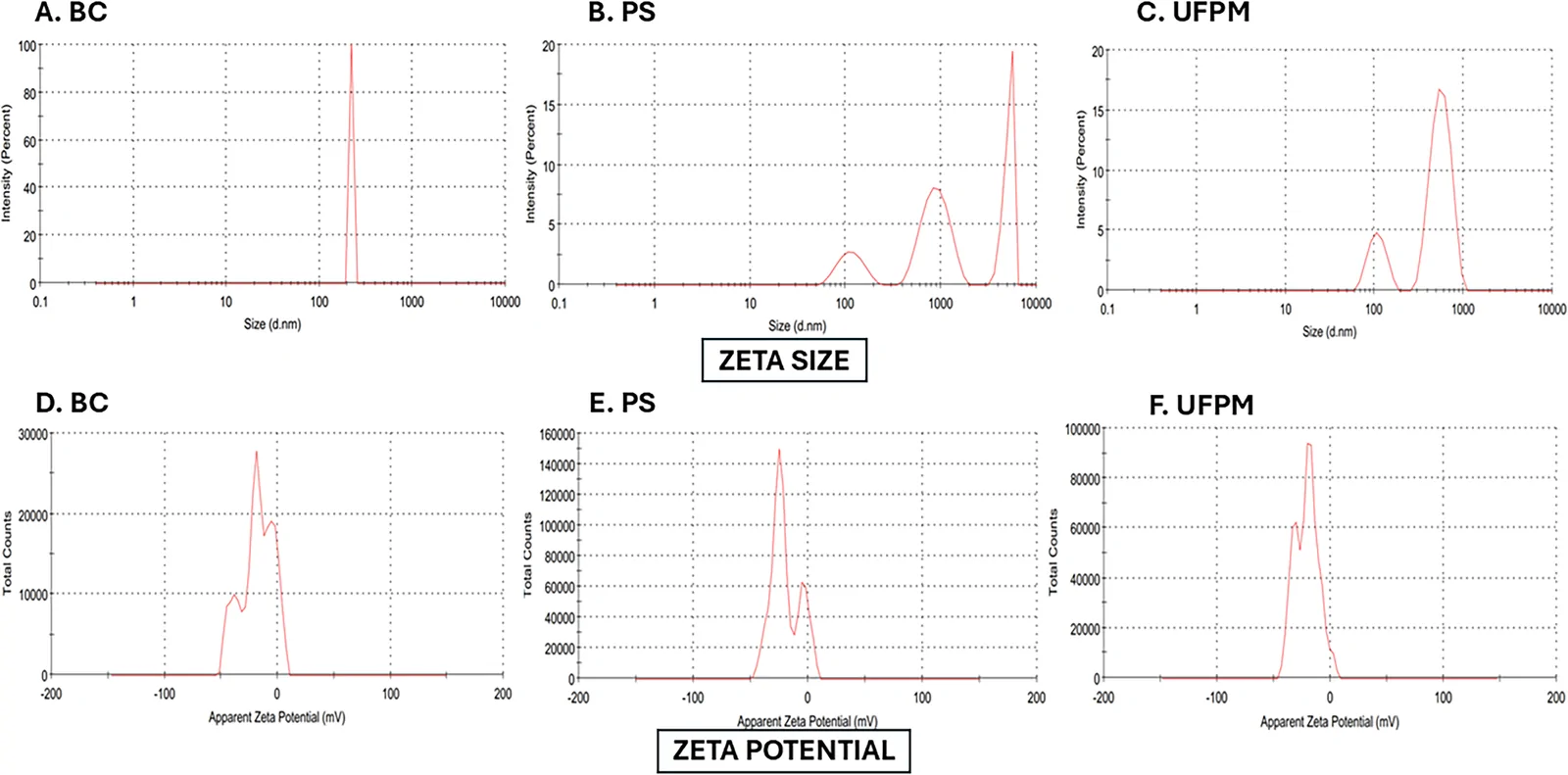
Physicochemical characterization of BC, PS-NPs, and UFPM. **(A–C)** Size distribution of black carbon (BC), polystyrene nanoplastics (PS-NPs), and ultrafine particulate matter (UFPM) based on dynamic light scattering (DLS). Black carbon had an average hydrodynamic diameter of 220.2 nm, PS-NPs had 566.2 nm, and ultrafine particulate matter showed heterogeneous distributions around 34.1, 566.1, and 903.0 nm. **(D–F)** Zeta-potential distributions of BC, PS-NPs, and UFPM, respectively, having apparent zeta potentials of -19.9, -16.3, and -26.1 mV.

These physicochemical properties may affect interactions between these nanoparticles and cells, such as dispersion in suspension, interaction with the plasma membrane, biomolecular corona formation, and particle uptake. Different hydrodynamic size profiles of BC and PS-NPs can affect their interaction with the plasma membrane. The larger hydrodynamic size of PS-NPs can result in different cell interactions. Similarly, this is true for UFPM, where heterogeneous particle-size distribution may affect cellular interaction depending on particle size. Surface charge properties may modulate these interactions by regulating electrostatic interactions between these particles and biomolecules associated with the cell membrane. However, because this experiment did not directly study the particle uptake mechanism, these relationships may reflect physicochemical determinants of differential interactions rather than direct evidence of the particle uptake pathway. Overall, DLS and zeta-potential analyses show that BC, PS-NPs, and UFPM exhibit distinct hydrodynamic and surface-charge properties. These physicochemical characteristics will help interpret PBMCs’ subsequent cellular response to these nanoparticles.

### 3.2 Flow-cytometric analysis demonstrates oxidative stress and mitochondrial membrane perturbation following nanoparticle exposure

Flow cytometric analysis also incorporated a 48 h incubation period to assess the time course of oxidative stress and mitochondria-associated fluorescence. We assessed fluorescence distributions relative to the respective non-incubated controls. Fluorescence distributions associated with CM-H_2_DCFDA fluorescence showed a gradual rightward shift among the 6, 12, 24, and 48 h exposure groups compared with their corresponding non-exposed control groups (**Supplementary Figure S1**) for BC, PS-NPs, and UFPM. BC revealed an apparent rightward shift in fluorescence distribution at 6 h that shifted further rightward at 12 h and 24 h, followed by a more pronounced fluorescence distribution in the 48 h group (**Supplementary Figure S1A**). PS-NPs also showed a similar temporal shift in fluorescence distribution, gradually shifting rightward at 6, 12, 24, and 48 h (**Supplementary Figure S1B**). Similarly, UFPM also showed a rightward shift in fluorescence distribution at 6 h, followed by rightward shifts at 12, 24, and 48 h (**Supplementary Figure S1C**). Thus, all three particulate exposures showed a general rightward fluorescence distribution pattern in CM-H_2_DCFDA.

CellROX profiles showed a similar trend across all three exposure groups (**Supplementary Figure S2**). BC caused rightward displacement of CellROX fluorescence distributions at 6, 12, 24, and 48 h compared with the control, with the 24- and 48 h distributions shifted even further toward higher fluorescence (**Supplementary Figure S2A**). PS-NPs caused a rightward shift in CellROX fluorescence distributions at 6, 12, 24, and 48 h, with the 24- and 48 h distributions shifted even further toward higher fluorescence (**Supplementary Figure S2B**). The same trend was observed for UFPM, which shifted CellROX fluorescence distributions to the right at 6, 12, 24, and 48 h, with the 24- and 48 h distributions shifted further right than the control (**Supplementary Figure S2C**). Thus, all three particles caused a similar direction of change in CM-H_2_DCFDA and CellROX fluorescence, but the extent of the shift varied between exposure groups.

Further analysis of the change in mitochondrial-associated fluorescence was conducted using the DiIC1 dye, along with SYTOX Blue staining (**Supplementary Figure S3**). BC, PS-NPs, and UFPM resulted in a rightward shift in DiIC1 fluorescence distributions compared with the control group, suggesting altered DiIC1 fluorescence after particulate exposure (**Supplementary Figures S3A–C**). The overlaid distribution profiles showed differences between the exposure groups, as the distributions of exposed cells extended further along the DiIC1 fluorescence axis than those of the control. SYTOX Blue staining revealed that most of the cells stayed in the SYTOX Blue-negative region in the presented profiles, while some of the SYTOX Blue-positive events were also present. Because the exposure times are plotted together in each figure, the representative bivariate plots suggest an alteration in the DiIC1 fluorescent signal associated with mitochondria, indicating altered DiIC1 fluorescence profiles following particulate exposure. Overall, flow cytometry revealed similar trends in fluorescent values for oxidative stress in BC, PS-NPs, and UFPM, as well as in mitochondrial DiIC1 fluorescence. Nonetheless, the differences in fluorescent values among BC, PS-NPs, and UFPM suggest variability in intensity and fluorescence.

### 3.3 Particle-specific temporal regulation of mitochondrial stress, bioenergetic, and inflammatory pathways

To evaluate the differential molecular responses generated by environmentally ubiquitous nanoparticles with chemically diverse properties, the temporal expression of genes implicated in mitochondrial stress signaling, oxidative stress, mitochondrial bioenergetics, and inflammatory signaling in PBMCs exposed to UFPM, BC, and PS-NPs for 6, 12, and 24 h (**Figure 2**) was investigated. Exposure of PBMCs to the three nanoparticles resulted in differential transcriptional remodeling among nanoparticles. Regarding the mitochondrial ISR (mtISR), gene expression of OMA1 and DELE1 was consistently upregulated after BC exposure, whereas UFPM and PS-NP exposures induced progressive and transient increases in gene expression, respectively. In addition, NRF2 and SOD2 transcript levels remained elevated in PBMCs exposed to BC, but UFPM and PS-NPs exposures resulted in delayed and reduced antioxidant responses, respectively. Mitochondrial adaptation and bioenergetic genes were also differentially regulated depending on the nanoparticles used. While UFPM induced HIF-1α at 12–24 h, it was not upregulated in cells treated with PS-NPs. In addition, PGC-1α was recovered from the suppression induced by UFPM, but PGC-1α was relatively constant in cells exposed to BC. BC and UFPM moderately upregulated TFAM, but PS-NPs induced comparatively higher TFAM levels after 24 h of exposure. By contrast, the mitochondrial respiratory gene ND1 remained stable after BC exposure, was progressively inhibited after PS-NPs exposure, and was restored after UFPM exposure. Likewise, the inflammatory response varied over time, with BC consistently increasing the expression of the inflammation-associated genes TNF-α and NLRP3. In contrast, UFPM showed transient expression of NF-κB, NF-κB p65, and TNF-α, peaking at 12 h, while NLRP3 expression was sustained. In contrast, PS-NPs induced the lowest inflammatory response, with reduced TNF-α expression and lower expression of NF-κB/NLRP3-associated genes. To determine whether the transcriptional alterations observed were functional consequences for the mitochondria, mitochondrial respiratory chain activities (Complexes I-V) were investigated after nanoparticle exposure (**Supplementary Figures S4**, **S5**). Changes in respiratory-chain complex activities were nanoparticle exposure-dependent and provided functional evidence complementary to the transcriptional findings.

**Figure 2:**
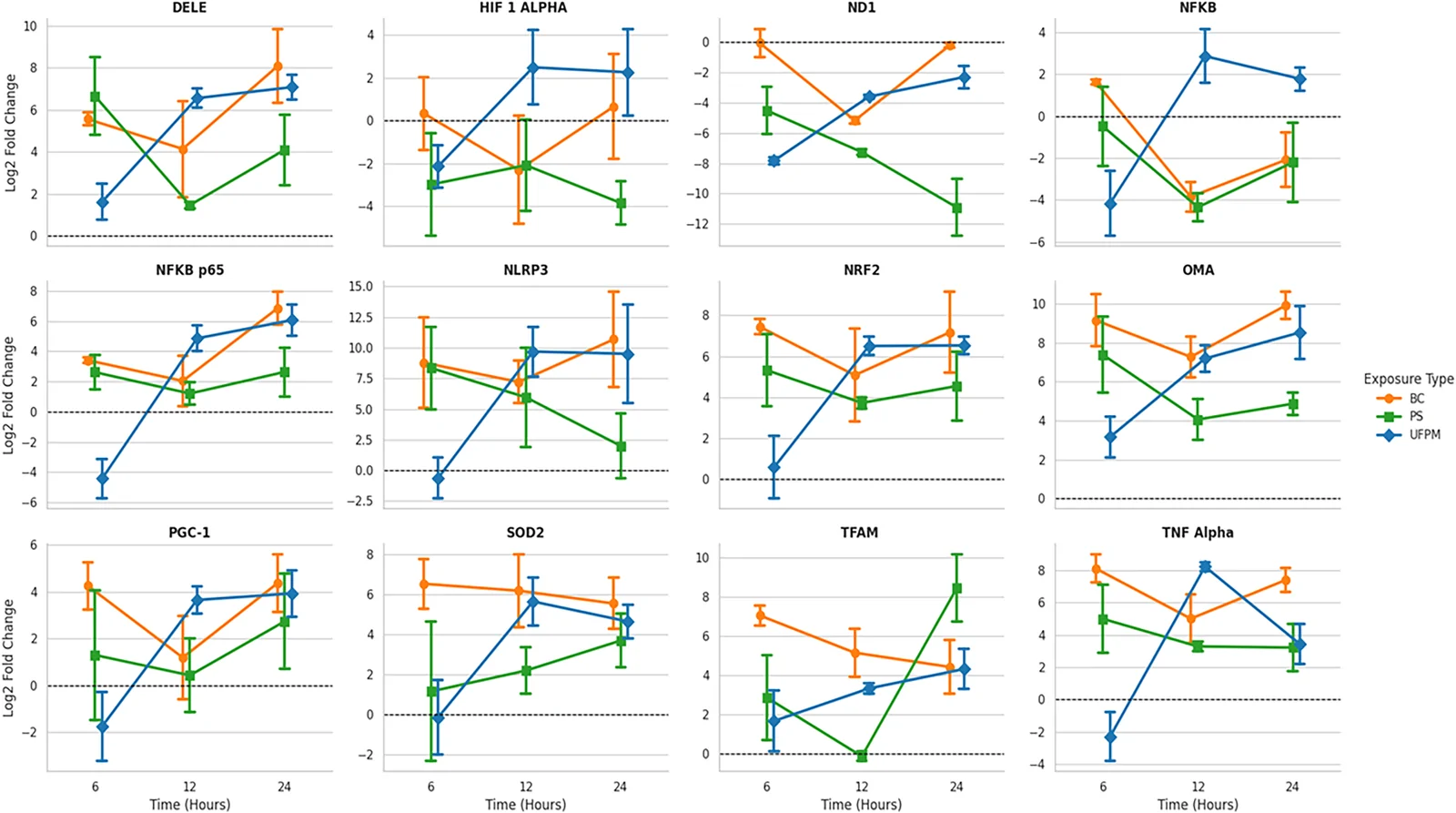
Time-dependent regulation of mitochondrial, inflammatory, and oxidative stress response gene expression following treatment with BC, PS-NPs, and UFPM. Time-dependent regulation of the expression of mRNAs for DELE, HIF-1α, ND1, NF-κB, NF-κB p65, NLRP3, NRF2, OMA1, PGC-1α, SOD2, TFAM, and TNF-α was examined at 6, 12, and 24 h post-treatment with black carbon (BC; orange), polystyrene nanoplastics (PS; green), and ultrafine particulate matter (UFPM; blue). We measured expression relative to the respective internal control gene and presented it as a log2 ratio compared with the untreated control sample, indicated by the horizontal dotted line at zero. Values are presented as mean ± SEM. The error bars show the standard error of the mean. The current time course experiment highlights time-dependent regulation of pathways related to mitochondrial stress response (DELE1-OMA1), hypoxia (HIF-1α), mitochondrial function (ND1, TFAM), oxidative stress (NRF2, SOD2), mitochondrial biogenesis (PGC-1α), and inflammation (NF-κB, NF-κB p65, NLRP3, and TNF-α).

In summary, comparisons among the three particulates revealed commonalities and differences in cellular responses. Although the particulates differ in their physicochemical attributes, UFPM, BC, and PS-NPs share common mitochondrial stress response pathways, including OMA1-DELE1-associated mitochondrial ISR, antioxidant response, mitochondrial energetic adaptation, and inflammatory signaling. Nevertheless, the magnitude and kinetics of these responses differed by particle type. UFPM caused the most comprehensive transcriptional remodeling over time, including rapid activation of adaptive and inflammatory responses. In contrast, BC produced the most sustained activation of ISR and inflammatory responses, along with mitochondrial oxidative stress. In addition, PS-NPs induced the weakest inflammatory responses but the most prominent regulation of mitochondrial adaptive and biogenesis-related genes, including TFAM, as well as persistent suppression of ND1. These results suggest that different nanoparticle types engage overlapping mitochondrial stress-signaling pathways while producing distinct bioenergetic and inflammatory responses.

### 3.4 Principal component analysis reveals exposure- and time-dependent transcriptional signatures

To assess the transcriptional relationships among the tested conditions (UFPM, BC, PS-NPs), we applied PCA to PBMCs exposed to UFPM, BC, and PS-NPs for 6, 12, and 24 h (**Figure 3**). Collectively, PC1 and PC2 accounted for 73.1% of the variation, with PC1 explaining 60.9% and PC2 12.2%, indicating that these two components explain most of the variation. Clustering by nanoparticle type separated the samples into distinct clusters, supporting particle-specific molecular response profiles. We also observed the progression of transcriptional alterations in samples exposed to UFPM, BC, and PS-NPs at 6, 12, and 24 h. Moreover, among all nanoparticle types, UFPM showed the largest area covered by PCA projection over time. BCs formed the smallest cluster with gradual movement in time. Samples from PS-NPs formed more dispersed clusters in PCA space than the others, indicating greater dispersion over time.

**Figure 3:**
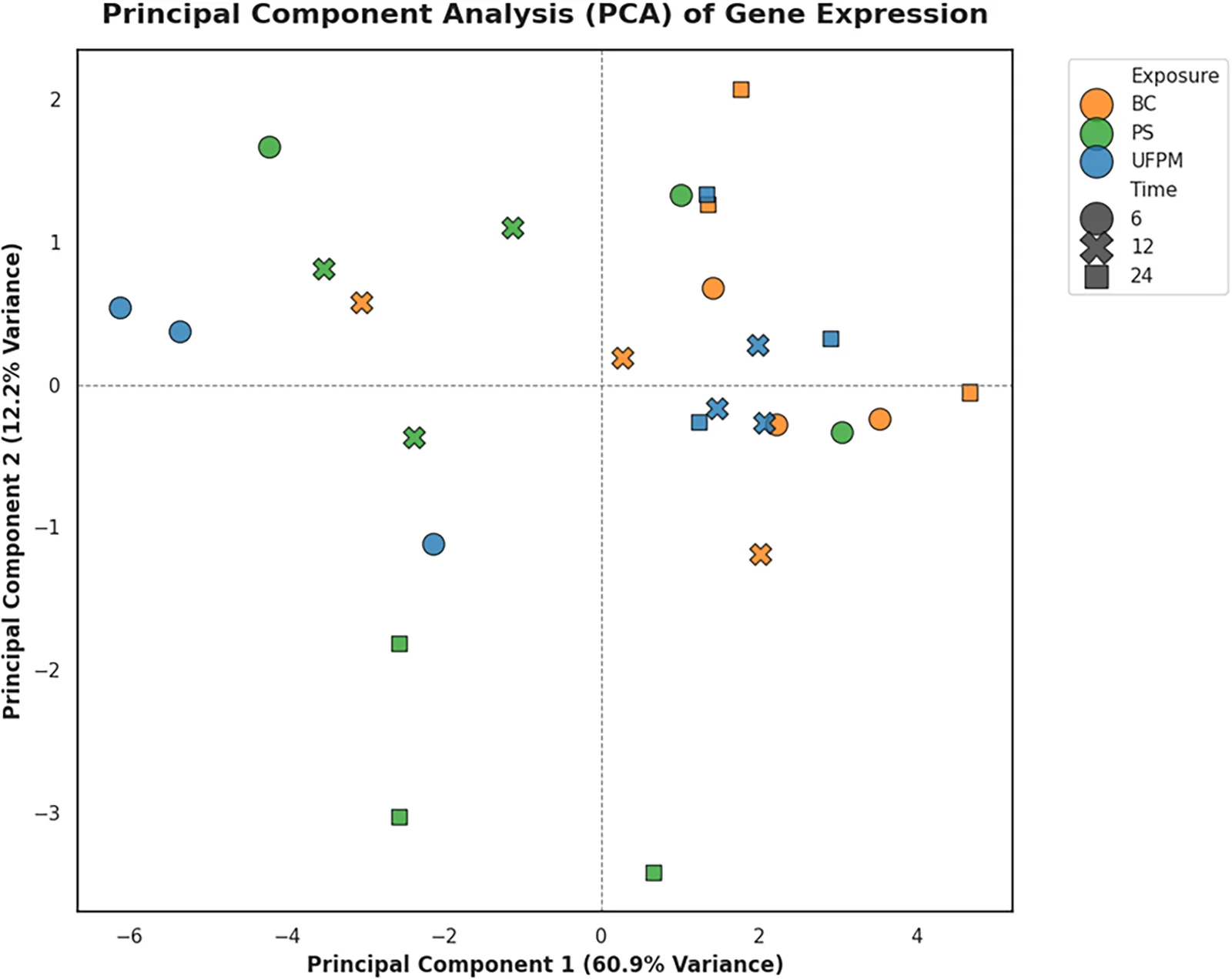
Principal component analysis (PCA) of transcriptional response upon exposure to BC, PS-NPs, and UFPM. A PCA was conducted using the normalized expression profiles of DELE1, HIF-1α, ND1, NF-κB, NF-κB p65, NLRP3, NRF2, OMA1, PGC-1α, SOD2, TFAM, and TNF-α following exposure to BC, PS-NPs, and UFPM for 6, 12, and 24 h. Color codes differentiate between types of exposures (BC - orange; PS - green; UFPM - blue), while different symbols differentiate between the time of exposure (circles - 6 h; crosses - 12 h; squares - 24 h). PC1 and PC2 account for 60.9% and 12.2% of the total variance, respectively.

### 3.5 Differential temporal regulation of FAPY following particulate exposure

To determine the kinetics of mitochondrial oxidative DNA damage and the DNA repair response, we compared FAPY expression patterns between particulate-exposed and untreated PBMCs following BC, PS-NPs, and UFPM exposure (**Figure 4**). Each nanoparticle type elicited distinct FAPY expression kinetics due to differences in exposure effects. While BC induced a gradual rise in FAPY expression in exposed cells, with mild up-regulation at 6 and 12 h and the highest expression at 24 h, the untreated control showed the opposite trend, with down-regulation throughout the experiment. In contrast, FAPY was generated by PS-NPs at 6 h and declined further at 12 and 24 h; however, it remained elevated above basal levels even at 24 h in the presence of the treated cells. UFPM exhibited another distinct time-dependent trend. FAPY expression was first downregulated at 6 h, nearly returned to baseline at 12 h, and peaked only after 24 h of exposure, providing evidence of delayed activation of the DNA repair response. In contrast, the untreated UFPM samples demonstrated continuous down-regulation. Thus, comparing FAPY expression across the three particulates revealed distinct regulatory kinetics depending on their nature. Although all three nanoparticle exposures altered FAPY expression, the timing and intensity of the response depend on the exposure’s physicochemical properties.

**Figure 4:**
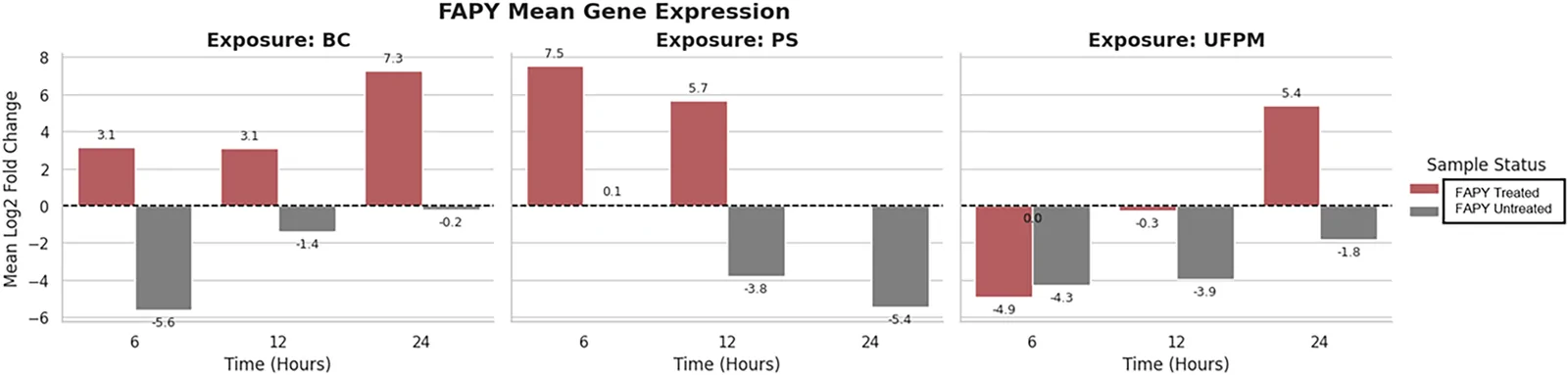
Time course of FAPY expression in treated and untreated cells exposed to BC, PS-NPs, and UFPM. Comparison of mean log2 fold change in FAPY expression between treated and untreated cells upon exposure to BC, PS-NPs, and UFPM for 6, 12, and 24 h was performed. The data shown represent mean gene expression values. Values above zero indicate up-regulation, while values below zero indicate down-regulation relative to the untreated control cells marked with a horizontal dashed line (0). Bars are grouped according to exposure type and sampling time to facilitate comparison of treatment-dependent temporal changes in mitochondrial gene expression.

### 3.6 Two-way ANOVA identifies exposure- and interaction-dependent regulation of mitochondrial stress pathways

We conducted a two-way ANOVA to assess the independent and interaction effects of nanoparticle exposure and exposure time on gene expression (**Figure 5**). Of the three factors considered, the exposure × time interaction appeared to have the strongest influence on the transcriptional profile, with significant interactions detected for ND1, NF-κB, NF-κB p65, TNF-α, NRF2, TFAM, DELE1, and OMA1 (all p < 0.05). The data suggest that transcriptional regulation of mitochondrial bioenergetics, oxidative stress, mtISR, and inflammatory signaling depends on the combined effect of nanoparticle type and exposure time, rather than on each factor individually. Separate main-effects analyses showed that exposure affected the transcriptional activity of the ND1, TNF-α, SOD2, and OMA1 genes, while time significantly affected only the NF-κB p65 and TFAM genes. In contrast, exposure and time showed no main effects for NLRP3, HIF-1α, and PGC-1α, suggesting that nanoparticle type or exposure time did not significantly explain their expression in this analysis. Thus, mitochondrial respiration (ND1), mtISR (OMA1-DELE1), antioxidant defense (NRF2-SOD2), mitochondrial biogenesis (TFAM), and inflammatory signaling (NF-κB/NF-κB p65 and TNF-α) show distinct associations with nanoparticle exposure, exposure time, and their interaction.

**Figure 5:**
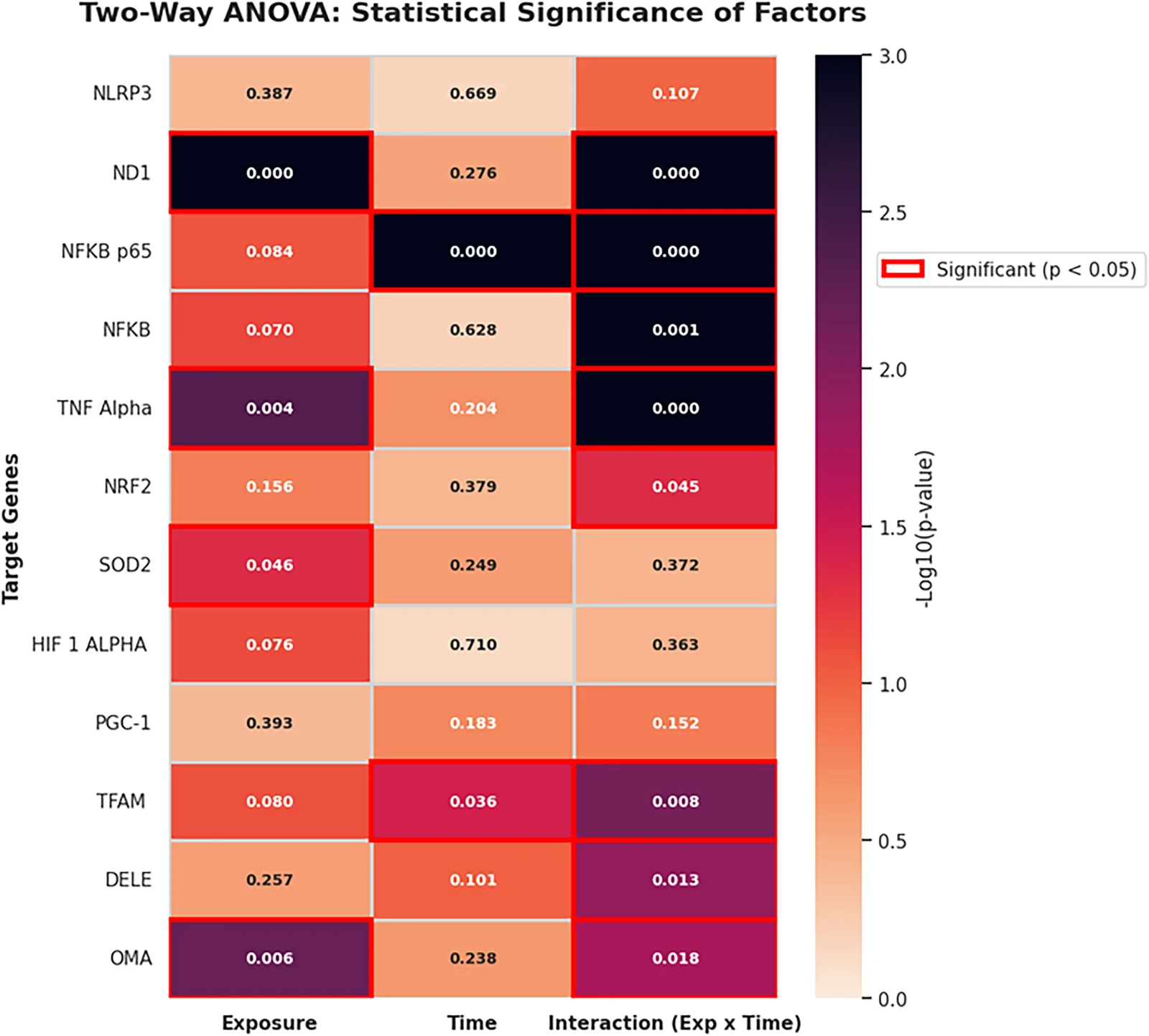
Two-way ANOVA reveals the impact of the interaction between exposure and time on the expression of mitochondrial stress-related genes, inflammatory genes, and oxidative stress-related genes. The heatmap shows the statistical significance of exposure, time, and their interaction on the expression of genes of interest, based on a two-way ANOVA. Color in the cells denotes −log_10_(p-value), where darker colors signify higher statistical significance. The values written in each cell are the corresponding p-values. Statistically significant impacts are red-framed (p<0.05). The test was conducted for NLRP3, ND1, NF-κB p65, NF-κB, TNF-α, NRF2, SOD2, HIF-1α, PGC-1α, TFAM, DELE1, and OMA1.

### 3.7 Hierarchical clustering reveals coordinated regulation of mitochondrial stress pathways

Hierarchical clustering was performed to examine relationships among exposure groups based on their interactions with genes linked to mitochondria, bioenergetics, oxidative stress, and inflammation (**Figure 6**). The heat map analysis revealed clusters of samples by nanoparticle type and exposure time, showing remodeling of transcriptional profiles with increasing exposure time. Generally, samples collected 24 h after exposure separated from the corresponding 6 h and 12 h samples and showed distinct expression profiles for stress-related genes. In contrast, early exposure groups had relatively low gene expression, whereas the 12 h samples had intermediate transcriptional profiles, corresponding to the transition from the initial adaptive response to mitochondrial stress. The gene dendrogram analysis also showed two major co-expression modules. First, the module included ND1, HIF-1α, and NF-κB genes that encode proteins involved in mitochondrial respiratory function, hypoxic adaptation, and inflammatory signaling, respectively. Module 2 included NLRP3, OMA1, NF-κB p65, PGC-1α, TFAM, SOD2, TNF-α, DELE1, and NRF2, indicating involvement of mtISR oxidative stress, antioxidant response, mitochondrial biogenesis, and inflammatory signaling. 24 h exposure to BC and UFPM showed increased expression of genes involved in mitochondrial stress and inflammation, namely OMA1, DELE1, NRF2, TNF-α, and NLRP3. Yet, the early-exposed groups showed less transcriptional activity. Overall, hierarchical clustering shows that nanoparticles influence transcription through functional gene groups rather than individual genes. Despite the different nanoparticles used, 24 h exposure triggered similar molecular mechanisms, namely mtISR, antioxidant response, mitochondrial adaptation, and inflammatory signaling.

**Figure 6:**
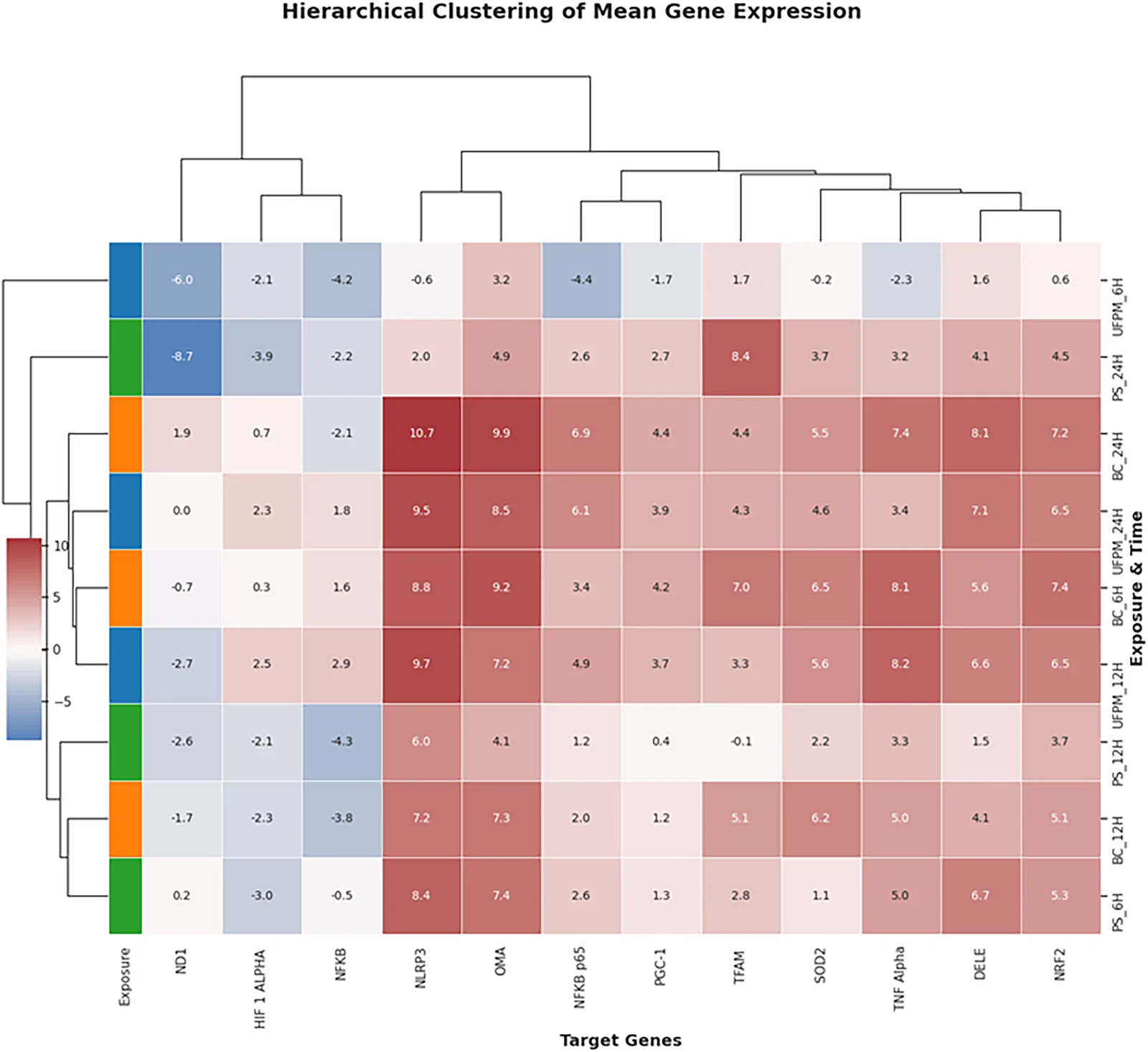
Hierarchical clustering of average gene expression profiles upon exposure to BC, PS-NPs, and UFPM. Heat map showing hierarchical clustering of average log_2_ fold-change values for genes involved in mitochondrial stress, inflammation, oxidative stress, hypoxia, and mitochondrial biogenesis in response to BC, PS-NPs, and UFPM at 6, 12, and 24 h. Row numbers indicate exposure time, while column numbers refer to the genes being studied (ND1, HIF-1α, NF-κB, NLRP3, OMA1, NF-κB p65, PGC-1α, TFAM, SOD2, TNF-α, DELE1, and NRF2). We performed hierarchical clustering using Euclidean distance and Ward’s linkage to identify similarities in gene expression. Color codes used in the cells are based on average log2 Fold changes; blue represents low expression, while red represents high expression.

### 3.8 Gene-to-gene correlation analysis reveals coordinated mitochondrial stress networks

We studied interactions among genes related to mitochondrial stress, oxidative stress, mitochondrial energetics, and inflammation under nanoparticle exposure using Pearson correlation analysis (**Figure 7**). The Pearson correlation test showed moderate to high correlations among genes involved in mitochondrial stress response, oxidative metabolism, bioenergetics, and inflammation. These correlations suggest coordinated changes in variable values but do not prove a causal relationship. We observed no negative correlations, suggesting that mitochondrial stress response, antioxidant protection, mitochondrial adaptation, and inflammatory responses varied in coordination with mitochondrial and oxidative-stress-related transcripts. We found positive correlations between genes involved in different biological pathways. Maximum correlations were found between TNF-α and NRF2 (r = 0.86), PGC-1α and SOD2 (r = 0.84), DELE1 and NRF2 (r = 0.84), NRF2 and NF-κB p65 (r = 0.83), DELE1 and NF-κB p65 (r = 0.82), and ND1 and OMA1 (r = 0.80). Moreover, OMA1, PGC-1α, NLRP3, TNF-α, SOD2, and NRF2 showed strong correlations. These results show substantial association among the mtISR, antioxidant response, mitochondrial biogenesis, and inflammation pathways.

**Figure 7:**
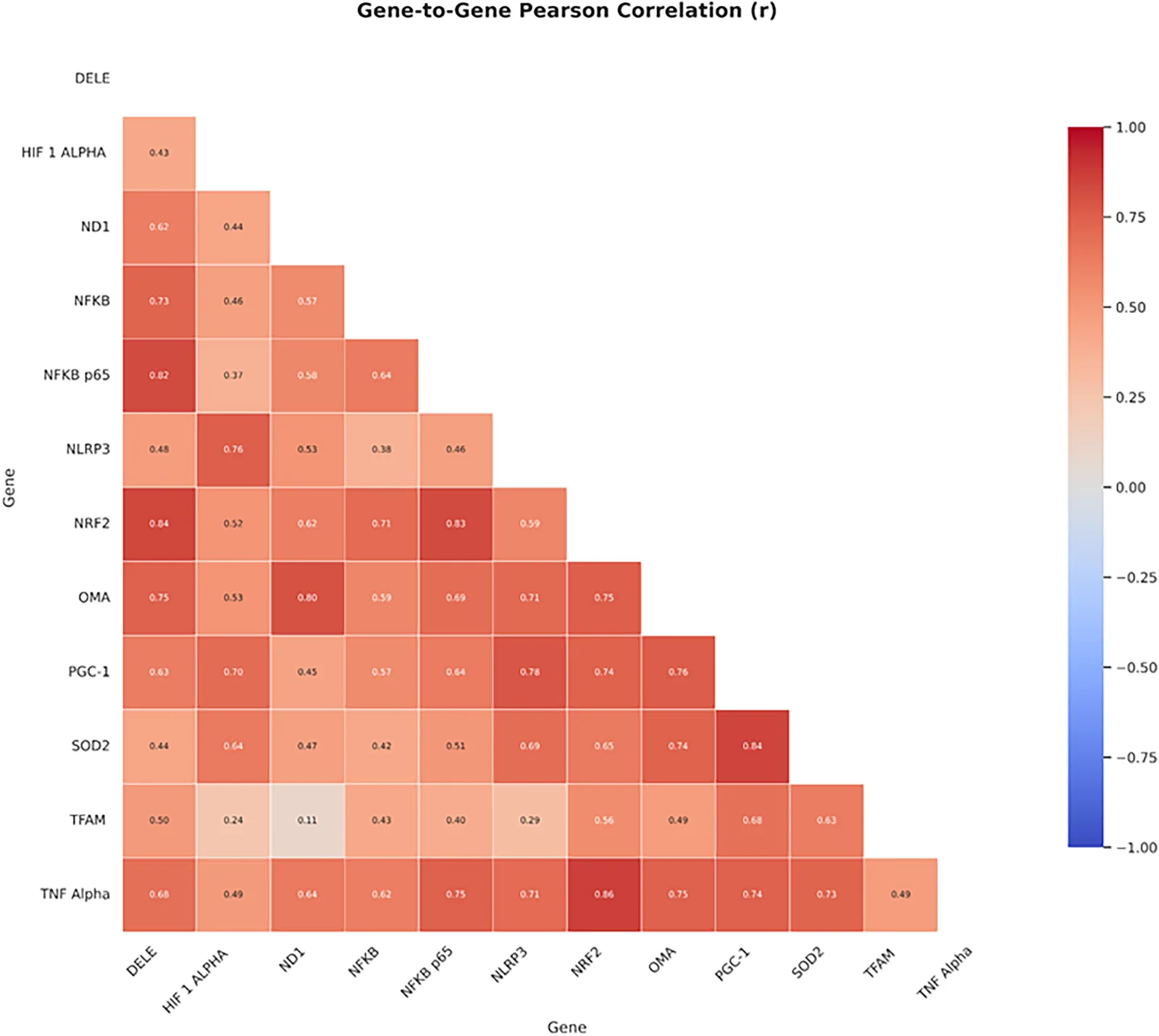
Pearson correlation between gene expression in mitochondrial stress, inflammatory, oxidative stress, and mitochondrial biogenesis pathways. Heat map showing Pearson correlation coefficients (r) between the expression levels of DELE1, HIF-1α, ND1, NF-κB, NF-κB p65, NLRP3, NRF2, OMA1, PGC-1α, SOD2, TFAM, and TNF-α across all exposure conditions and different time points. The bottom part of the matrix shows the Pearson correlation coefficients for each gene pair. It shows the degree and type of correlation from -1 (negative correlation; blue) to +1 (positive correlation; red) according to the color scale. We used Pearson’s correlation to assess transcriptional synchrony among genes involved in mitochondrial quality control, oxidative stress response, inflammation, hypoxia, and mitochondrial biogenesis after BC, PS, and UFPM treatment.

### 3.9 Shared variance analysis supports coordinated regulation of mitochondrial stress pathways

Moreover, we computed the coefficient of determination (R²) to quantify shared variance among gene expression levels and assess shared transcriptional variability of mitochondrial stress response pathways (**Figure 8**). As with the Pearson correlation analysis, some genes involved in mitochondrial stress, oxidative stress, and inflammation showed considerable shared variance, indicating that a substantial proportion of the observed variation in the two variables was shared across the analyzed conditions. The genes with the greatest shared variance included TNF-α and NRF2 (R² = 0.74), PGC-1α and SOD2 (R² = 0.71), DELE1 and NRF2 (R² = 0.70), NRF2 and NF-κB p65 (R² = 0.68), DELE1 and NF-κB p65 (R² = 0.67), as well as ND1 and OMA1 (R² = 0.64). Nonetheless, the correlation between OMA1, PGC-1α, NLRP3, TNF-α, and SOD2 suggests interconnectivity among pathways involved in mitochondrial ISR regulation, antioxidative responses, energy production, and inflammation. In contrast, TFAM showed low correlations with genes including ND1 (R² = 0.01), HIF-1α (R² = 0.06), and NLRP3 (R² = 0.09). Overall, the shared-variance analysis showed a well-integrated network of mitochondrial stress response pathways, while also indicating that certain pathway components, such as TFAM, were independent of other pathways under nanoparticle-induced cell stress.

**Figure 8:**
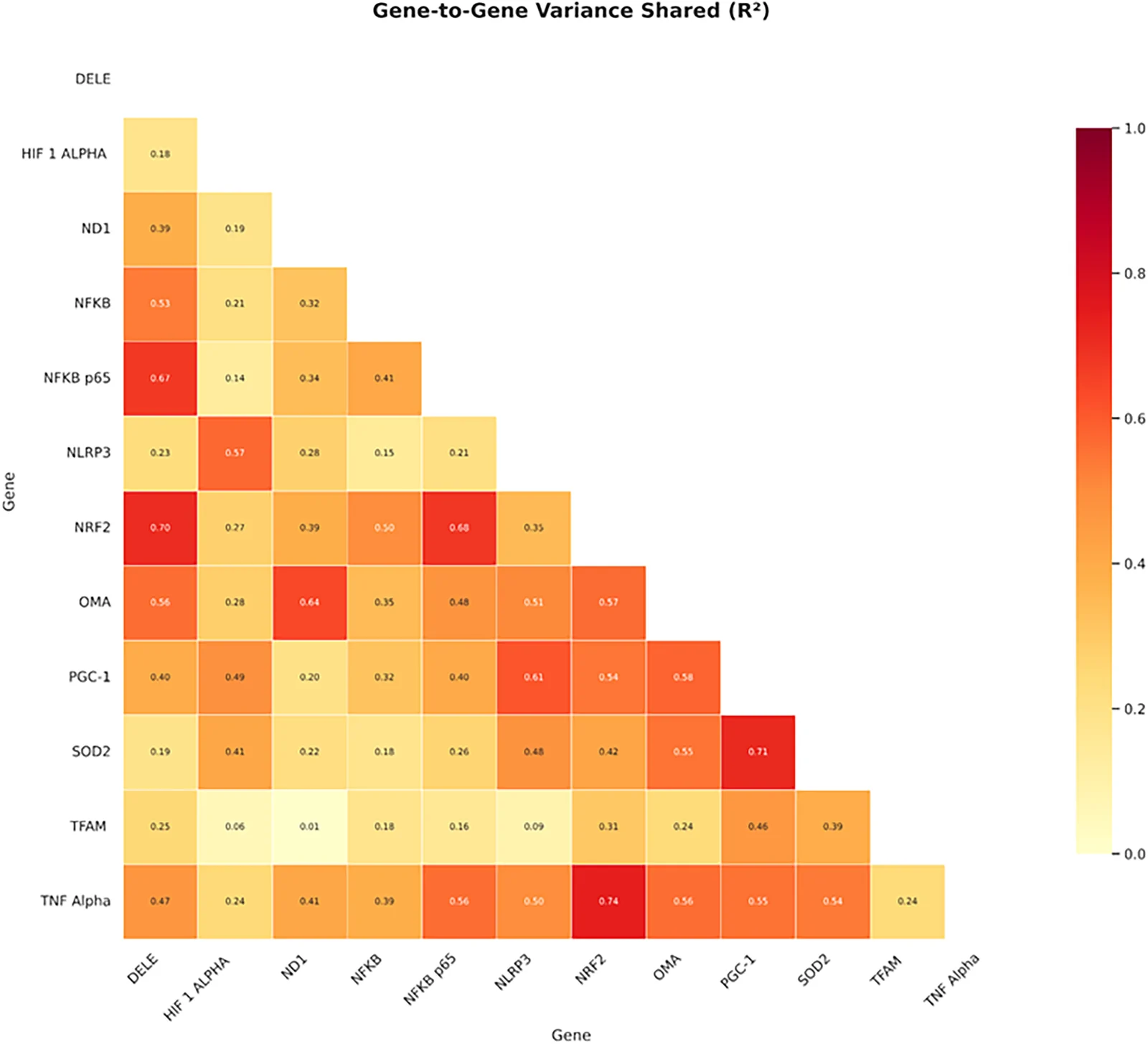
Shared variance (R²) among genes related to mitochondrial stress, inflammatory signaling, oxidative stress, and mitochondrial biogenesis. Heat map showing the pairwise coefficients of determination (R2) obtained using Pearson correlation analysis of the expression patterns of DELE1, HIF-1α, ND1, NF-κB, NF-κB p65, NLRP3, NRF2, OMA1, PGC-1α, SOD2, TFAM, and TNF-α at all exposure conditions and time points. R² values for each gene pair are shown in the lower triangle of the matrix and reflect the amount of shared variance in the gene expression profile. Numbers within each square show the R² values, while the color scale shows the extent of shared variance, where the light-yellow color represents 0 (low level of shared variance), whereas the dark red color indicates 1 (high level of shared variance). The study aimed to assess the degree of overlap in the transcriptional variation among genes related to mitochondrial quality control, hypoxia, oxidative stress, inflammation, and mitochondrial biogenesis after exposure to BC, PS-NPs, and UFPM.

### 3.10 Pairwise significance analysis supports coordinated mitochondrial stress networks

We performed statistical significance analysis to assess the strength of gene-gene relationships using correlation and variance analyses (**Figure 9**). As shown in the Pearson correlation matrix, most interactions among genes involved in mitochondrial stress, oxidative stress, bioenergetics, and inflammatory pathways were statistically significant (p < 0.05). Some of these associations were significant (p < 0.001) and included genes such as DELE1, OMA1, NRF2, PGC-1α, SOD2, TNF-α, NF-κB, and NF-κB p65. The findings point towards coordinated regulation of the mtDNA, antioxidant defense, mitochondrial adaptation, and inflammation-related pathways following nanoparticle exposure. At the same time, some genes, including TFAM, showed fewer significant interactions with other genes; namely, HIF-1α, ND1, and NLRP3 were not significantly associated with TFAM, and the interaction between HIF-1α and NF-κB p65 was not statistically significant. These data suggest that although mitochondrial biogenesis is part of the general stress response network, some genes responsible for mitochondrial genome integrity demonstrate greater transcriptional independence than genes responsible for mtDNA, oxidative stress, and inflammatory signaling. Overall, the significance analysis supports the stress response network established using clustering and correlation analyses.

**Figure 9:**
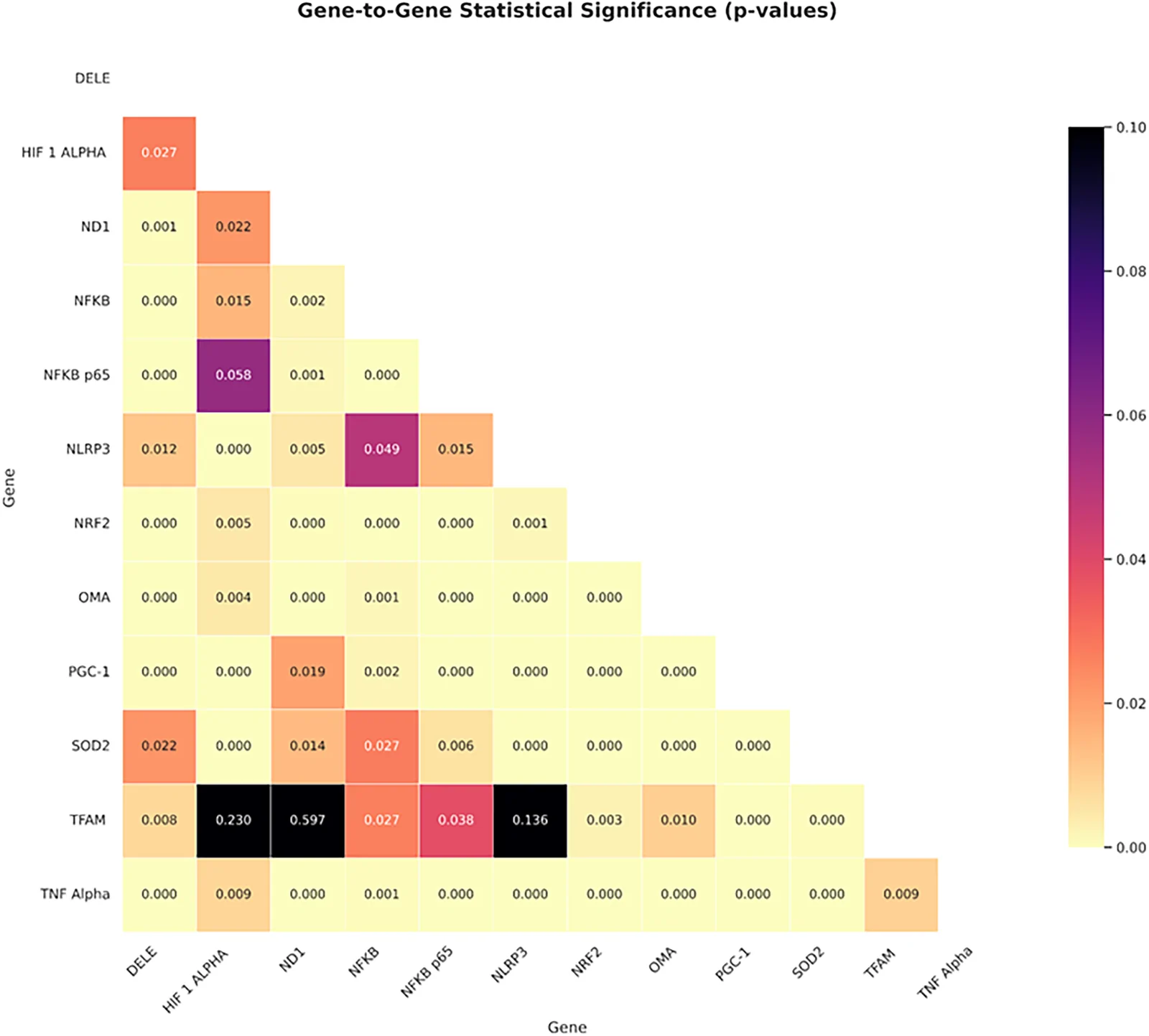
Statistical significance of pairwise gene-gene correlations in mitochondrial stress, inflammation, oxidative stress, and mitochondrial biogenesis pathways. Heat map indicating the p-values resulting from the pairwise Pearson correlation analysis on the expression profiles of DELE1, HIF-1α, ND1, NF-κB, NF-κB p65, NLRP3, NRF2, OMA1, PGC-1α, SOD2, TFAM, and TNF-α under all exposure conditions and all time points. The lower triangular portion of the matrix shows the p-value for each gene pair, with values presented within each cell. Color intensity denotes p-value significance, with lighter colors indicating low p-values (high statistical significance), and vice versa, according to the color scale provided. In this study, we analyzed the statistical significance of interactions involving transcriptional regulation of genes involved in mitochondrial quality control, hypoxia signaling, oxidative stress, inflammation, and mitochondrial biogenesis following BC, PS-NPs, and UFPM treatment.

### 3.11 Integrative correlation analysis links mitochondrial stress pathways with inflammatory cytokine secretion

To assess associations between transcriptional alterations and inflammatory cytokine secretion, we used Pearson’s correlation analysis to compare the expression levels of mitochondrial stress-related genes with cytokine concentrations measured by ELISA (**Figure 10**). Cytokine-specific correlation patterns have been revealed. IL-6 and IL-8 showed correlation with the expression levels of genes related to mitochondrial stress, oxidative stress, and inflammation, whereas IL-1β showed low or even negative correlations. TNF-α secretion showed moderate correlations with several mitochondrial stress-induced genes. Among the cytokines considered, IL-6 had the strongest correlations with mitochondrial stress pathways, including high positive correlations with HIF-1α and NF-κB (r = 0.73), together with moderate correlations with DELE1, NLRP3, OMA1, PGC-1α, NRF2, and ND1. Similarly, IL-8 had strong correlations with genes participating in mtDNA, oxidative stress response, and inflammation, including high positive correlations with NF-κB p65 (r = 0.72), PGC-1α (r = 0.58), SOD2 (r = 0.52), NLRP3 (r = 0.49), and NRF2 (r = 0.49). In contrast, TNF-α release showed moderate positive correlations with ND1, HIF-1α, Mitogen ND1, NLRP3, OMA1, and SOD2, whereas IL-1β showed only weak correlations with most genes and negative correlations with several mitochondria-specific proteins, especially TFAM (r = -0.48). Altogether, the integrative transcriptional and cytokine analysis indicates the close relationship between transcriptional activation of mitochondrial stress pathways and cytokine secretion upon nanoparticle treatment. Genes involved in mitochondrial ISR (OMA1-DELE1), antioxidant response (NRF2-SOD2), hypoxic adaptation (HIF-1α), bioenergetics (ND1), and inflammatory signaling (NF-κB/NF-κB p65 and NLRP3) correlated selectively with IL-6 and IL-8, which showed the strongest associations with the mitochondrial stress-related transcriptional responses examined. Conversely, the relatively weak correlations of TFAM with all analyzed cytokines, especially IL-1β and TNF-α, suggest that mitochondrial biogenesis and DNA maintenance are regulated independently of acute cytokine secretion. These results provide integrated transcriptional and cytokine evidence linking mitochondrial dysfunction to downstream inflammation in response to environmentally relevant nanoparticles with distinct chemical compositions.

**Figure 10:**
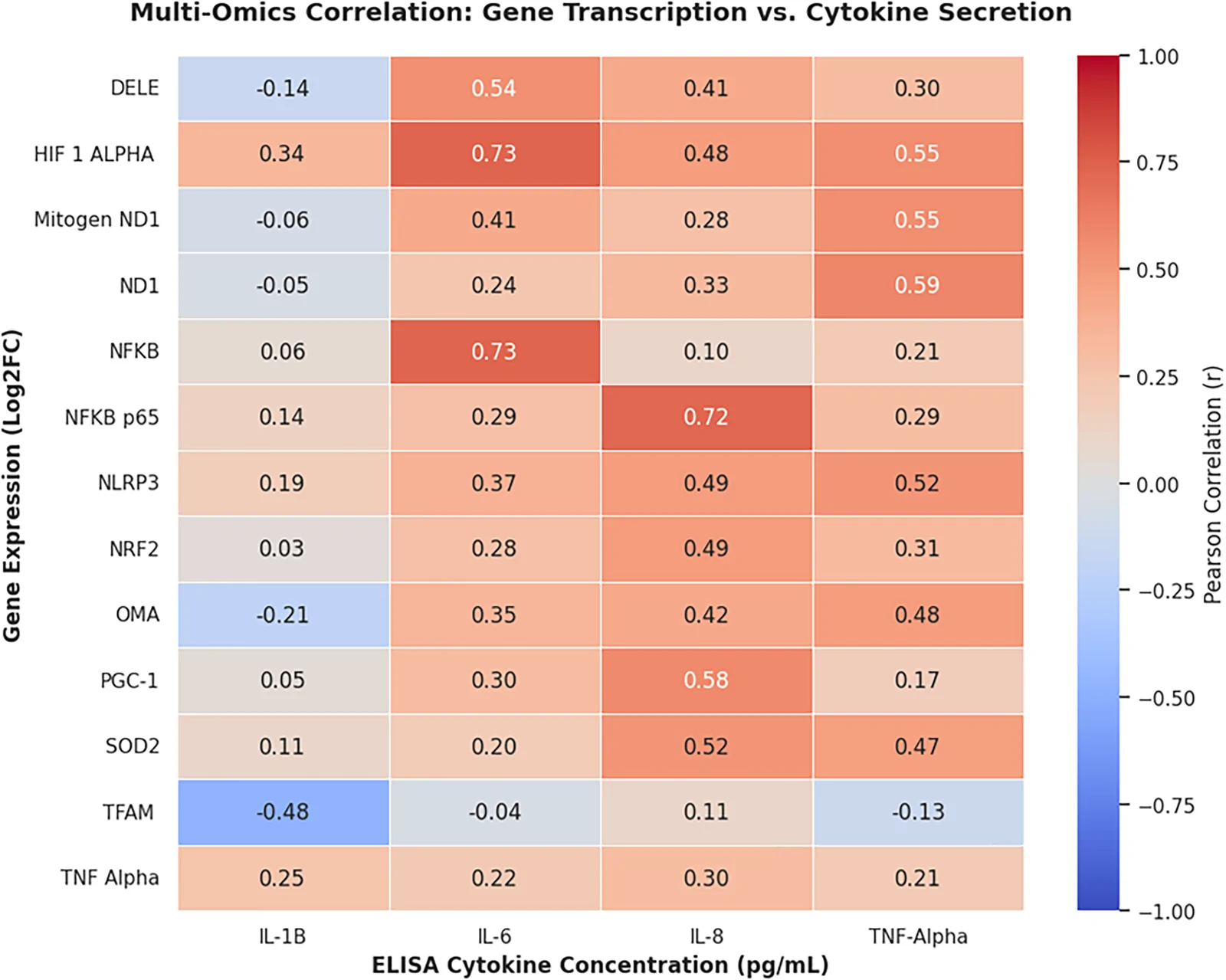
Correlation of gene transcription and cytokine secretion in response to airborne particulate pollutants. Heat map depicting Pearson correlation coefficients (r) of the relative expression of genes related to mitochondrial stress, inflammation, oxidative stress, and mitochondrial biogenesis and the level of cytokines measured using ELISA. Log2-transformed fold changes in gene expression for DELE1, HIF-1α, Mitogen ND1, ND1, NF-κB, NF-κB p65, NLRP3, NRF2, OMA1, PGC-1α, SOD2, TFAM, and TNF-α were correlated with the levels of IL-1β, IL-6, IL-8, and TNF-α at all exposure conditions and time points. Each cell shows the Pearson correlation coefficient (r), and the color scale indicates the degree of correlation, ranging from -1 (negative correlation; blue) to +1 (positive correlation; red). This holistic approach investigated the correlation between transcription and cytokine secretion in response to BC, PS-NPs, and UFPM.

Together, these studies suggest that exposure to different environmental nanoparticles elicits a dynamically regulated but conserved mitochondrial stress response. Although gene expression levels and dynamics differed, UFPM, BC, and PS-NPs showed overlapping responses involving the mtDNA (OMA1-DELE1), oxidative stress response (NRF2-SOD2), mitochondrial metabolic regulation (HIF-1α-PGC-1α-TFAM-ND1), and inflammatory signaling (NF-κB, NLRP3, and TNF-α). Using systems biology approaches such as PCA, hierarchical clustering, correlation, shared variance, and transcript and cytokine associations, we formed a clear picture of a connected network between mitochondrial function and cytokine secretion. Whereas BC caused sustained mitochondrial stress and an inflammatory response, UFPM caused the most dynamic transcriptional reprogramming, and PS-NPs showed relatively greater mitochondrial adaptation and comparatively weaker inflammatory responses. Together, these findings indicate a shared mitochondrial stress-adaptation framework associated with environmental nanoparticle immunotoxic responses, with distinct particle-specific molecular signatures.

## 4 Discussion

This study shows that different nanoparticles affect mitochondrial function similarly, despite their distinct chemical compositions. We analyzed the following nanoparticles for similar impacts in human PBMCs: UFPM, BC, and PS-NPs. Although individual studies have discussed the molecular effects of UFPMs, BC, and PS-NPs on mitochondria (10, 11, 18), comparative studies on this topic remain scarce. Although all three nanoparticles showed some similarities in stress markers, stress-pathway activity within mitochondria appears to differ. This reflects the unique characteristics of each nanoparticle: UFPM induces adaptation, BC induces mitochondrial dysfunction and inflammation, and PS-NPs induce bioenergetic changes. Our results further support the emerging idea that mitochondria are pivotal environmental stress sensors that integrate oxidative, metabolic, and inflammatory signals into a coordinated stress response rather than responding separately to different pollutants (16, 17, 36). Moreover, the physicochemical properties account for the differential cell responses to the three particulates. The hydrodynamic size and zeta-potential of BC, PS-NPs, and UFPM differed. The aforementioned parameters are known to affect the interaction of the particles with the cells, thus leading to different kinetics of mitochondrial stress response. From the DLS results, we found heterogeneity in particle size distribution and aggregation, particularly for UFPMs and PS-NPs. However, because we studied particle properties in suspension without cells, we could not rule out changes due to aggregation, corona formation, and cell-particle interactions. Furthermore, this study did not include a separate endotoxin analysis.

Our results suggest that mitochondrial redox dysregulation is an early cellular response associated with UFPM and BC exposure, whereas PS-NPs may follow slightly different pathways to the same outcome. Redox-sensitive genes were upregulated in response to nanoparticulate exposure, though kinetics differed depending on the nature of the nanoparticulate. While UFPM caused a quick and transient response of the antioxidant defense system, this is likely due to a high level of reactivity of its surface and the presence of transition metal ions and quinones, which facilitate redox cycling and Fenton-type reactions (3, 4). Unlike BC exposure, BC was associated with the sustained expression of NRF2, implying oxidative stress signaling triggered by continuous ROS generation from combustion products such as polycyclic aromatic hydrocarbons; this work aligns with previous findings that BC exposure leads to persistent inflammation and mitochondrial dysfunction, resulting in mitochondria-related oxidative stress (5, 10, 14). Finally, PS-NPs cause mitochondria-associated stress through nanoparticle interactions *in vivo* rather than through reactions on their surfaces (11). The positive correlations between NRF2-TNF-α (r = 0.86), NRF2-DELE1 (r = 0.84), and PGC-1α-SOD2 (r = 0.84) imply a relationship between oxidative stress and mitochondria. Remarkably, PS-NPs induced downstream effects related to mitochondrial stress without a strong oxidative stress response, suggesting that mitochondrial disturbances can also be triggered through other pathways, including changes in mitochondrial dynamics and interactions. These findings are consistent with oxidative stress being one of several processes associated with mitochondrial stress responses.

Flow cytometry studies functionally validated the oxidative stress response. Changes in CM-H2DCFDA and CellROX fluorescence indicated shifts in cellular redox balance, while DiIC1 staining showed a simultaneous change in mitochondrial membrane potential (**Supplementary Figures S1**–**S3**). These results complement the transcriptional data and confirm the role of oxidative and mitochondrial stress in the response to particulate exposure. Beyond eliciting oxidative stress, our flow cytometry data also show additional disruptions in mitochondrial membrane potential caused by nanoparticle exposure. This phenotype is consistent with our finding that nanoparticle exposure affects the expression profiles of genes linked to bioenergetic reprogramming. Our finding that HIF-1α, PGC-1α, TFAM, ND1, and Complex I-V activity are affected is consistent with mitochondria’s dynamic response to oxidative stress to maintain energy homeostasis (27, 37, 38). However, the degree of remodeling depends on the nanoparticle type. UFPM activated HIF-1α, regulated PGC-1α levels, and increased TFAM expression, which helped adapt to oxidative stress (39, 40). In BC, HIF-1α activation and a low respiration rate indicated that mitochondrial dysfunction was adaptive (4, 10, 14, 24). The non-challenging PS-NPs increased TFAM expression, consistent with previous studies on nanoplastics’ effects on mitochondria (11). The correlation between ND1 and OMA1 (r = 0.80), as well as between PGC-1α and SOD2 (r = 0.84), indicates that respiration, mitochondrial production, and antioxidants are all linked in a coordinated mechanism. In consequence, although all three nanoparticle types evoke a similar mitochondrial stress response, the chemical nature of the particles determines whether mitochondrial bioenergetic adaptation occurs successfully (18).

Another important observation is the increased expression of the OMA1 and DELE1 genes in response to all three nanoparticle types, suggesting that mtISR is part of the cellular adaptive stress response. Recently, the OMA1-DELE1 pathway has been recognized as an important regulator of mitochondrial protein quality control; however, its contribution to the particle-induced mitochondrial stress is poorly understood (16, 17, 41). Despite differences in oxidative stress dynamics, UFPM, BC, and PS-NPs treatment increased OMA1 and DELE1 transcript expression, indicating an OMA1-DELE1-associated mitochondrial stress response. While BC led to sustained expression of OMA1 and DELE1, as well as oxidative stress and respiratory dysfunction, the temporary responses to UFPM and the small increase in gene expression from PS-NPs treatment suggest possible differences in the intensity and duration of the mitochondrial stress responses. Therefore, although OMA1-DELE1-associated responses occurred in response to all three particle exposures, their magnitude and persistence differed significantly. Moreover, the strong positive correlations between DELE1 and NRF2 (r = 0.84) and between ND1 and OMA1 (r = 0.80) also suggest a close relationship among oxidative stress, respiratory impairment, and mitochondrial surveillance. This study supports an OMA1-DELE1-associated mtDNA induced by nanoparticles in PBMCs and suggests that this pathway may be incorporated into the mitochondrial stress response network (18, 42). However, because this work has not studied OMA1-dependent DELE1 processing, downstream ISR signaling, or functional perturbation of OMA1 or DELE1, the transcriptional changes and correlations described above do not reflect direct activation or the role of the complete OMA1-DELE1 pathway. Further research involving protein validation and targeting of OMA1/DELE1 will be necessary to determine whether this axis has any functional impact on particle-induced mitochondrial and inflammatory responses.

Further, our results suggest that inflammatory activation is associated with, and may occur downstream of, mitochondrial stress rather than representing an entirely independent response to nanoparticle exposure. Consistent with our proposed model, NF-κB, NF-κB p65, TNF-α, and NLRP3 are primarily expressed during later exposure phases. Moreover, the findings show that inflammation activation is linked to mitochondrial stress and can be viewed as a coordinated response to nanoparticle exposure rather than an independent one. The delayed appearance of NF-κB, NF-κB p65, TNF-α, and NLRP3 in response to PS-NPs exposure further supports this argument, and this temporal correlation does not mean that mitochondrial stress causes an inflammatory response (20).

In contrast to previous studies comparing one particle class at a time, our current study provides evidence that all three nanoparticles activate a mitochondrial stress response program in the same experimental setting (10, 43, 44). Furthermore, the simultaneous stimulation of NLRP3, NF-κB, and TNF-α signaling pathways implies the priming function of the inflammasome mediated by the danger signal emitted by mitochondria, consistent with past research demonstrating the importance of UFPM and PS-NPs in NLRP3 activation via the mitochondrial oxidative stress pathway (11, 45–47). In addition, the strong correlation between TNF-α and NRF2 (r = 0.86), as well as between HIF-1α and NLRP3 (r = 0.76), supports an association among inflammation, oxidative stress, and metabolic alterations rather than independent reactions. Overall, the data discussed above support a model in which persistent mitochondrial stress may favor inflammatory signaling in exposed cells.

The multivariate statistical approach strengthens the proposed comparative framework by showing coordinated patterns in nanoparticle-mediated mitochondrial stress responses. PCA is common in nanoparticle toxicology to integrate multiple dimensions of physicochemical and biological properties and obtain a particle-specific response signature (34). PCA is also used to characterize environmental nanoparticles and aerosols to define particle variation patterns and exposure signatures (33, 35). The percentage of variance explained by the first two Principal Components (PC1 and PC2) through PCA was 73.1% (PC1 – 60.9%, PC2 – 12.2%). This suggests that oxidative stress control, mitochondrial remodeling, and inflammation lead to variability. At the molecular level, mitochondrial adaptation drives the high variability seen in the UFPM group. BC particles were placed on the positive PC1 axis, which showed mitochondrial and inflammatory damage. The PS-NPs location is highly variable due to mitochondrial adaptation in the presence of low inflammation. Hierarchical clustering also shows that exposure duration contributed substantially to transcriptional organization, alongside nanoparticle-specific effects, suggesting that although the nanoparticles have distinct chemical characteristics, they become similar in effect because they trigger a common mitochondrial stress response. Correlation and shared-variance analyses show high intercorrelations among NRF2, DELE1, PGC-1α, SOD2, ND1, OMA1, TNF-α, and NLRP3, presenting coordinated variance in mitochondrial stress, antioxidants, bioenergy, and inflammation markers. Altogether, these systems analyses suggest that particle physicochemical properties are linked to differences in the kinetics and degree of mitochondrial responses observed in the different exposure scenarios (18, 48–52).

Our results not only support results from other studies on how UFPM, BC, and PS-NPs can cause mitochondrial dysfunction but also build on existing knowledge (9–11). Unlike prior work, which has studied mitochondria after exposure to individual nanoparticle classes across various experimental models, this study enables direct comparison of these particles under the same experimental conditions to investigate both common and particle-specific effects on the mitochondrial system. The particles converged at the level of mitochondrial stress-response pathways but differed in the intensity, kinetics, and persistence of mitochondrial adaptation. At the level of mitochondrial stress-response pathways, the particles showed differences only in terms of the intensity and dynamics of mitochondrial adaptation, thus confirming and expanding upon the previously observed mitochondrial dysfunction and mitoepigenetic regulation caused by exposure to particulate matter (4, 14, 18). Using human PBMCs in the current study increases the translational relevance of the findings, since these circulating immune cells are a readily available proxy for systemic exposure and can serve as biomonitoring tools in environmental monitoring efforts. The study has the following limitations. First, applying a common mass concentration does not mean UFPM, BC, and PS-NPs have equal particle numbers, particle surface area, or cellular particle load. Variations in particle size distribution, aggregation, morphology, and surface characteristics can affect the dose particles deliver to cells and can thus lead to particle-dependent biological effects. Normalizing for particle numbers or surface areas, along with measuring cellular effective doses, will be critical for further comparative analysis. Therefore, the current results should be considered as a response to a common mass-based dose rather than equal particle-number or surface-area doses. Second, this study cannot determine chronic exposure and accumulation in organs or tissues. Third, while oxidative stress and mitochondrial damage were analyzed using flow cytometry, the OMA1-DELE1 pathway and mitochondrial metabolism have not been investigated. Future studies should combine *in vivo* experiments with single-cell transcriptomics, spatial transcriptomics, phosphoproteomics, and mitochondrial metabolomics in order to confirm the physiological relevance of the proposed pathway (50, 53, 54). Another limitation is the heterogeneity of immune cells in the PBMC model. PBMCs comprise several immune cell types; however, the current experimental design does not allow measurement of the response by individual cell type. Thus, any change in the expression of inflammatory genes, cytokines, oxidative stress, and mitochondrial function is associated with PBMCs, not with specific immune cell types. From an applied perspective, the regulatory coordination among OMA1, DELE1, NRF2, PGC-1α, TFAM, and ND1 suggests that these genes associated with the mtISR may be potential biomarkers that require validation in subsequent studies (5, 55). Overall, these results suggest mitochondrial stress adaptation as a common mechanism underlying nanoparticle toxicity and pave the way for developing mechanism-based biomarkers of environmental exposure. Based on these results and the molecular responses observed, we propose a conceptual framework in which chemically distinct nanoparticle exposures are associated with particle-specific upstream disruptions that ultimately converge on an integrated mitochondrial stress response network. Mitochondrial adaptation varies across UFPM, BC, and PS-NPs exposures (**Figure 11**).

**Figure 11:**
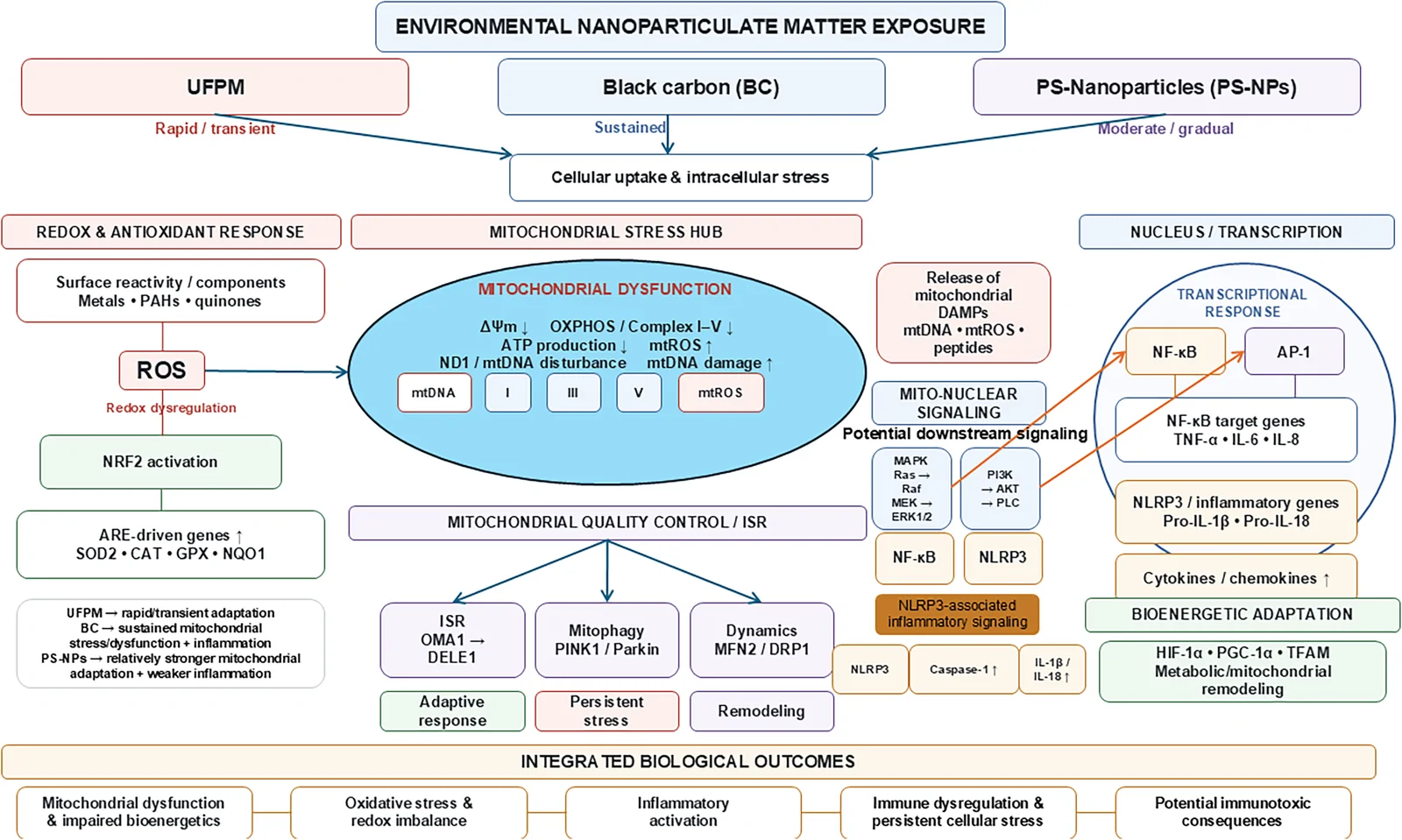
Proposed comparative model of the relationship between particle-specific mitochondrial adaptation to stress and inflammatory response. Differently composed UFPM, BC, and PS NPs induce particle-specific stress response pathways that involve mitochondrial, oxidative stress, energetic, and inflammatory pathways. Variability in the magnitude, kinetics, and persistence of the response leads to distinct molecular and immunotoxic outcomes.

## 5 Conclusion

This study provides evidence that different nanoparticles with varying physicochemical properties (UFPM, BC, and PS-NPs) exhibit overlapping mitochondrial stress responses. By directly comparing different environmental nanoparticles under similar experimental conditions in human PBMCs, we show that oxidative stress, mitochondrial bioenergetic reprogramming, OMA1-DELE1-associated mitochondrial integrated stress responses, and inflammation form a network of cellular responses whose magnitude and kinetics vary with nanoparticle physicochemical properties. Based on these results, mitochondrial dysfunction is correlated with alterations in redox homeostasis, mitochondrial quality control, metabolic reprogramming, and inflammatory signaling. In addition, our comparative systems analysis showed that OMA1 and DELE1 levels were positively correlated with mitochondrial stress and inflammation parameters, suggesting the OMA1-DELE1 pathway may be involved in nanoparticle-mediated responses and warranting further investigation. Although the correlation between stress-related mitochondrial, oxidative, bioenergetic, and inflammatory measures indicates a cellular response to particulate matter exposure, it does not prove any cause-and-effect relationship between these pathways. We also found a complex network of functionally associated biomarkers, including NRF2, PGC-1α, TFAM, ND1, OMA1, DELE1, TNF-α, NLRP3, and mitochondrial respiratory Complex I-V. Overall, our results show that environmental nanoparticles are associated with mitochondrial stress-adaptive responses, with unique signatures of mitochondrial dysfunction that could serve as biomarkers in future studies. However, further investigations involving protein-level validation and functional perturbations of OMA1 and DELE1 will be needed to establish the role of this axis in particle-induced mitochondrial and inflammatory responses.

## Data Availability

The raw data supporting the conclusions of this article will be made available by the authors, without undue reservation.
