# Supplementary material for: Conserved and particle-specific mitochondrial stress responses drive immunotoxicity of ultrafine particulate matter, black carbon, and polystyrene nanoplastics
Source: Front Immunol. 2026 Oct 19;17:1993305. doi: 10.3389/fimmu.2026.1993305 (PMC13724557; doi:10.3389/fimmu.2026.1993305)
Supplement: Supplementary file 1 [file Supplementaryfile1.docx]

**Supplementary methods**

**2.1 Sample collection and lymphocyte isolation**

Blood samples from healthy adults were drawn via venipuncture with approval from the Institutional Ethics Committee of the ICMR-National Institute for Research in Environmental Health, Bhopal, India. Blood sampling procedures followed the ethical guidelines. The samples were transported to the laboratory at 2-8 °C within 2-3 h of collection. PBMCs were separated by density gradient centrifugation technique using HiSep™ LSM 1077 (HiMedia Laboratories Pvt. Ltd., Mumbai, India) as per the manufacturer's protocol. The PBMCs were first washed twice with sterile PBS solution and then were pelleted by centrifugation. PBMCs were cultured in the presence of UFPM, BC, or PS-NPs. PBMCs were harvested after 6, 12, and 24 h. PBMCs that were not subjected to any treatment and maintained under similar culture conditions served as controls. The cell pellets were collected after each time point and preserved at -80 °C for further analysis (Mishra et al., 2008; Bhargava et al., 2020).

**2.2 Characterization of particulates**

The physicochemical properties of UFPM, BC, and PS-NPs were examined by measuring particle size, distribution, aggregation, and surface charge. Samples were prepared in distilled water and mixed thoroughly before analysis. Particle size and polydispersity index (PDI) were recorded using dynamic light scattering, and zeta potential was measured by electrophoretic light scattering using a Zetasizer Nano ZS. All measurements were carried out three times, and values are presented as mean ± standard deviation (Gurjar et al., 2024).

**2.3 Treatment and exposure of UFPM, BC, and PS**

UFPM, BC, and PS stock suspensions were prepared in 1X PBS. Dilutions were prepared to achieve a final concentration of 1 µg/mL (Bhargava et al., 2017; Bhargava et al., 2019; Salinas et al., 2020; Adamiak et al., 2025; Mishra et al., 2026). The same dose was applied to all three particulates to facilitate direct comparison under similar conditions. This dose was chosen to study early oxidative stress, mitochondrial dysfunction, and inflammation without causing unnecessary cell death. The cells were exposed to the nanoparticulates for 6, 12, and 24 hours under a humidified environment of 5% CO_2_. The control group included unexposed cells. After exposure to each particulate for the respective time duration, the cells were pelleted by centrifugation at 4,500 × g for 10 min. The cells were then washed twice in 1X PBS to remove any unbound particles. Cells were stored at -80 °C for further processing (Mishra et al., 2010; Salinas et al., 2020; Adamiak et al., 2025).

**2.4 Evaluation of oxidative stress and mitochondrial dysfunction**

Genomic DNA was extracted from control and treated PBMC samples (UFPM, BC, and PS-NPs) using the PureLink™ Genomic DNA Mini Kit (Invitrogen, Thermo Fisher Scientific, USA) following the manufacturer’s protocol. We assessed oxidatively damaged genomic DNA by detecting oxidized purine bases, such as 8-oxodeoxyguanosine, using an Fpg digestion assay, as described previously (Gupta et al., 2019; Bunkar et al., 2020). We evaluated cellular oxidative-stress-related fluorescence using the CM-H₂DCFDA labeling method. We analyzed cellular fluorescence by flow cytometry. mtROS-related fluorescence was quantified by CellROX™ Deep Red Reagent (500 nM) treatment for 1 hour at 37°C. ΔΨm was assessed after 6, 12, 24, and 48 h of exposure. Flow cytometry results were analyzed with respect to corresponding non-exposed control groups at each exposure time point. Flow-cytometric analysis first identified the cellular population based on forward- and side-scatter characteristics, then excluded debris and non-cellular events. For the DiIC1/SYTOX Blue assay, we excluded SYTOX Blue-positive, membrane-compromised events from the final analysis and evaluated DiIC1 fluorescence within the remaining cellular population.

**2.5 Gene expression analysis of oxidative stress-related markers**

RNA isolation was followed by cDNA synthesis. Relative quantitative PCR was conducted using primers for the NRF2 and SOD2 genes. Normalization was performed using a housekeeping gene, and quantification was calculated using the 2^^-∆∆CT^ method. The experiment was conducted in triplicates (Mishra et al., 2022).

**2.6 Assessment of mitochondrial functional markers**

RNA was isolated from the samples and subjected to reverse transcription into cDNA. The qRT-PCR assay was used to assess the expression levels of HIF-1α, PGC-1α, and TFAM genes. Expression levels were normalized against the internal gene, and the relative expression levels were calculated using the 2^^-∆∆CT^ method. All analyses were done in triplicates.

**2.7 Assessment of mitochondrial stress signaling**

Mitochondrial stress signaling was assessed by analyzing the expression of DELE1 and HRI using one-step quantitative real-time PCR.  Relative gene expression was calculated by the 2^^-ΔΔCT^ method after normalization with the housekeeping gene (Soni et al., 2025a,b).

**2.8 Evaluation of mitochondrial complex activity assay**

PBMCs were exposed to UFPM, BC, and PS (1µg/mL) for 6, 12, and 24 h. Cells were collected by centrifugation at 4,500 × g for 10 min. Total protein was extracted from control and treated cells. Mitochondrial electron transport chain (ETC) complex activities were measured using MitoCheck Complex Activity Assay Kits (Cayman Chemical, Ann Arbor, MI, USA) according to the manufacturer's instructions. Absorbance readings were taken using a Spark® multimode microplate reader (Tecan, Männedorf, Switzerland) (Soni et al., 2025a, b). ETC complex activities were normalized to the total protein content of the corresponding sample before comparison among treatment groups.

**2.9 Immunotoxicity and inflammatory response assessment**

**2.9.1 Cytokine quantification**

Supernatants from cultures treated with UFPM, BC, and PS-NPs, as well as control cultures, were obtained. They were then centrifuged and stored in the deep freezer at -80°C for future analysis. The concentrations of IL-6, IL-8, and TNF-α in each sample were analyzed using Human GENLISA™ ELISA Kits (KRISHGEN BioSystems, Cerritos, CA, USA). Absorbance of each sample was determined at 450 nm using a Spark® multimode microplate reader (Tecan, Männedorf, Switzerland). The amount of each cytokine was determined using its calibration curve. High cytokine levels in treated samples were interpreted as inflammation (Jain et al., 2011).

**2.9.2 Transcriptional analysis of NF-κB pathway genes**

Total RNA from both control and treated PBMCs was isolated using the TRIzol™ reagent (Life Technologies, Thermo Fisher Scientific, Waltham, MA, USA). The total RNA concentration and purity were assessed. The first-strand cDNAs were synthesized from total RNA by using the iScript™ cDNA synthesis kit (Bio-Rad Laboratories, Hercules, CA, USA). The RELA and NFKB1 mRNA expression levels were quantified using the qRT-PCR method. The mRNA expression levels were normalized to an internal reference gene (Mishra et al., 2022).

**2.9.3 Assessment of NLRP3 inflammasome expression profiles**

Total RNA was extracted from both control and treated PBMCs. RNA concentration and purity were determined spectrophotometrically. cDNA was synthesized from equal amounts of RNA using a reverse transcription kit. The relative expression of the NLRP3 gene was estimated using qPCR with gene-specific primers. The relative expression was normalized to the internal gene using the 2^^-ΔΔCT^ method. IL-1β expression in culture supernatants was estimated by ELISA. Culture supernatants after exposure to particles were centrifuged to remove cell debris and stored at -80 °C for further analysis. IL-1β levels were measured using a human IL-1β ELISA kit according to the manufacturer's protocol. The absorbance was recorded at 450 nm using a Spark® multimode microplate reader (Tecan, Männedorf, Switzerland). The concentration of cytokines was determined from the standard curve (Li et al., 2025).

**2.10 Statistical analysis**

All calculations were done in Python. Donor-derived experimental samples were considered the biological replicates and the statistical units. When technical replicates were available, we measured them three times using qPCR and ELISA, averaged the technical replicates before statistical analysis, and did not treat them as independent biological observations. Gene expression data are represented as mean log2 fold-change ± SEM of biological replicates. For datasets with the factors nanoparticle type (UFPM, BC, and PS-NPs) and time (6, 12, and 24 h), we used two-way ANOVA to analyze the main effects of nanoparticle treatment, treatment time, and their interaction. When effects were significant (p < 0.05), we performed post-hoc pairwise comparisons with appropriate corrections for multiple hypothesis testing. We calculated the Pearson correlation coefficient (r), coefficient of determination (R²), and corresponding p-values from the biological-replicates data. We analyzed biological-replicate data matrices using principal component analysis, hierarchical clustering, and K-means clustering. Correlations between gene-expression measurements and ELISA-derived cytokine levels were calculated across the measured exposure conditions and time points using biological-replicate data.

**References:**

1. Adamiak, K., Sidoryk-Węgrzynowicz, M., Dąbrowska-Bouta, B., Sulkowski, G., & Strużyńska, L. (2025). Primary astrocytes as a cellular depot of polystyrene nanoparticles. *Scientific Reports*, *15*(1), 6502. https://doi.org/10.1038/s41598-025-91248-w
2. Bhargava, A., Kumari, R., Khare, S., Shandilya, R., Gupta, P. K., Tiwari, R., ... & Mishra, P. K. (2020). Mapping the mitochondrial regulation of epigenetic modifications in association with carcinogenic and noncarcinogenic polycyclic aromatic hydrocarbon exposure. International Journal of Toxicology, 39(5), 465-476. https://doi.org/10.1177/1091581820932875.
3. Bhargava, A., Shukla, A., Bunkar, N., Shandilya, R., Lodhi, L., Kumari, R., Gupta, P. K., Rahman, A., Chaudhury, K., Tiwari, R., Goryacheva, I. Y., & Mishra, P. K. (2019). Exposure to ultrafine particulate matter induces NF-κβ mediated epigenetic modifications. Environmental Pollution, 252(Pt A), 39-50. https://doi.org/10.1016/j.envpol.2019.05.065.
4. Bhargava, A., Tamrakar, S., Aglawe, A., Lad, H., Srivastava, R. K., Mishra, D. K., Tiwari, R., Chaudhury, K., Goryacheva, I. Y., & Mishra, P. K. (2017). Ultrafine particulate matter impairs mitochondrial redox homeostasis and activates phosphatidylinositol 3-kinase mediated DNA damage responses in lymphocytes. *Environmental Pollution*, *234*, 406–419. https://doi.org/10.1016/j.envpol.2017.11.093
5. Bunkar, N., Sharma, J., Chouksey, A., Kumari, R., Gupta, P. K., Tiwari, R., Lodhi, L., Srivastava, R. K., Bhargava, A., & Mishra, P. K. (2020). Clostridium perfringens phospholipase C impairs innate immune response by inducing integrated stress response and mitochondrial-induced epigenetic modifications. *Cellular Signalling*, *75*, 109776. https://doi.org/10.1016/j.cellsig.2020.109776
6. Gupta, P., Bhargava, A., Kumari, R., Lodhi, L., Tiwari, R., Gupta, P. K., ... & Mishra, P. K. (2019). Impairment of mitochondrial-nuclear cross talk in lymphocytes exposed to landfill leachate. Environmental health insights, 13, 1178630219839013. https://doi.org/10.1177/1178630219839013.
7. Gurjar, V., Nazeer, N., Bhargava, A., Soni, N., Kaur, P., Tiwari, R., & Mishra, P. K. (2024). Deep learning-enabled nanophotonic test leveraging poly-L lysine-tethered carbon quantum dots to assess the risk of cardiovascular disease. *Microchemical Journal*, *207*, 112164. <https://doi.org/10.1016/j.microc.2024.112164>
8. Jain, D., Pathak, N., Khan, S., Raghuram, G. V., Bhargava, A., Samarth, R., & Mishra, P. K. (2011). Evaluation of cytotoxicity and anticarcinogenic potential of Mentha leaf extracts. International journal of toxicology, 30(2), 225-236. https://doi.org/10.1177/1091581810390527.
9. Kaur, P., Soni, N., Gurjar, V., Nazeer, N., Chouksey, A., Tiwari, R., Rajan, A., Ratre, P., Bhargava, A., Srivastava, R., & Mishra, P. (2025). Mitochondrial epigenetic alterations induced by biomass smoke exposure and their role in Age-Related Breast Cancer Risk: a Machine Learning-Based Predictive study. *Asian Pacific Journal of Cancer Prevention*, *26*(11), 4069–4078. <https://doi.org/10.31557/apjcp.2025.26.11.4069>
10. Li, D., Li, H., Wang, Z., Huang, Y., Zhao, C., Zhang, H., Zhao, Y., Zhu, N., Tang, X., Jin, Y., & Zhou, D. (2025). Time- and dose-dependent activation of the NLRP3 and MyD88/NF-κB pathways by Trueperella pyogenes membrane vesicles in bovine endometrial epithelial cells. *Veterinary Research*, *56*(1), 155. <https://doi.org/10.1186/s13567-025-01587-9>
11. Li, M., Li, Q. Z., Zhao, Y., & Gao, X. (2025). Recent advances in machine learning models for predicting toxicity of inorganic nanoparticles. Chem & Bio Engineering, 2(11), 647. https://doi.org/10.1021/cbe.5c00048.
12. Mishra, P. K., Khan, S., Bhargava, A., Panwar, H., Banerjee, S., Jain, S. K., & Maudar, K. K. (2010). Regulation of isocyanate-induced apoptosis, oxidative stress, and inflammation in cultured human neutrophils: isocyanate-induced neutrophils apoptosis. *Cell biology and toxicology*, *26*(3), 279-291.https://doi.org/10.1007/s10565-009-9127-9.
13. Mishra, P. K., Kumari, R., Bhargava, A., Bunkar, N., Chauhan, P., Tiwari, R., Shandilya, R., Srivastava, R. K., & Singh, R. D. (2022). Prenatal exposure to environmental pro-oxidants induces mitochondria-mediated epigenetic changes: a cross-sectional pilot study. *Environmental Science and Pollution Research*, *29*(49), 74133–74149. <https://doi.org/10.1007/s11356-022-21059-3>
14. Mishra, P. K., Panwar, H., Bhargava, A., Gorantla, V. R., Jain, S. K., Banerjee, S., & Maudar, K. K. (2008). Isocyanates induces DNA damage, apoptosis, oxidative stress, and inflammation in cultured human lymphocytes. Journal of Biochemical and Molecular Toxicology, 22(6), 429-440. https://doi.org/10.1002/jbt.20260.
15. Mishra, P. K., Rajan, A. K., Chouksey, A., Gurjar, V., Pathak, A., Aglawe, A., ... & Srivastava, R. K. Polymer-Specific Nanoplastics Trigger Oxidative Stress, Inflammatory Activation, and Mitoepigenetic Remodeling: In Vitro and In Silico Evidence for MT-ND6-Mediated Mitochondrial Complex I Perturbation. Frontiers in Toxicology, 8, 1916884. https://doi.org/10.3389/ftox.2026.1916884.
16. Ramaye, Y., Dabrio, M., Roebben, G., & Kestens, V. (2021). Development and Validation of Optical Methods for Zeta Potential Determination of Silica and Polystyrene Particles in Aqueous Suspensions. *Materials (Basel, Switzerland)*, *14*(2), 290. <https://doi.org/10.3390/ma14020290>
17. Salinas, M. E., Gutiérrez, D. A., Varela-Ramírez, A., & Garza, K. M. (2020). Continuous exposure to low doses of ultrafine black carbon reduces the vitality of immortalized Lung-Derived cells and activates senescence. *Journal of Toxicology*, *2020*, 1–13. https://doi.org/10.1155/2020/5702024
18. Soni, N., Chouksey, A., Gurjar, V., Gupta, K., Srivastava, R. K., Nema, R. K., Tiwari, R., & Mishra, P. K. (2025b). Mitochondrial epigenetic remodeling and integrated stress response induced by microcystin-LR: Experimental evidence and preliminary PP2A structural targeting. *Toxicon*, *269*, 108931. <https://doi.org/10.1016/j.toxicon.2025.108931>
19. Soni, N., Kaur, P., Gurjar, V., Bhargava, A., Tiwari, R., Chouksey, A., Srivastava, R. K., & Mishra, P. K. (2025a). Unveiling the interconnected dynamics of mitochondrial Dysfunction associated with Age-Related Cardiovascular Risk: a Cross-Sectional Pilot study. *Cureus*, *17*(4), e82961. <https://doi.org/10.7759/cureus.82961>.
20. Wang, X. Z., Yang, Y., Li, R., Mcguinnes, C., Adamson, J., Megson, I. L., & Donaldson, K. (2014). Principal component and causal analysis of structural and acute in vitro toxicity data for nanoparticles. Nanotoxicology, 8(5), 465-476. https://doi.org/10.3109/17435390.2013.796534.
